# Identification of Breed-Specific SNPs of Danish Large White Pig in Comparison with Four Chinese Local Pig Breed Genomes

**DOI:** 10.3390/genes15050623

**Published:** 2024-05-14

**Authors:** Xudong Wu, Decai Xiang, Wei Zhang, Yu Ma, Guiying Zhao, Zongjun Yin

**Affiliations:** 1 Anhui Provincial Key Laboratory of Livestock and Poultry Product Safety Engineering, Institute of Animal Husbandry and Veterinary Medicine, Anhui Academy of Agricultural Sciences, Hefei 230001, China; 2 Yunnan Academy of Animal Husbandry and Veterinary Sciences, Kunming 650224, China; 3 Anhui Provincial Laboratory of Local Animal Genetic Resource Conservation and Bio-Breeding, College of Animal Science and Technology, Anhui Agricultural University, Hefei 230036, China; 4 College of Animal Science and Technology, Yunnan Agricultural University, Kunming 650201, China

**Keywords:** Large White pig, genome, breed-specific, functional analysis

## Abstract

Genetic variation facilitates the evolution, environmental adaptability, and biodiversity of organisms. Danish Large White (LW) pigs have more desirable phenotypes compared with local Chinese pigs, which have difficulty adapting to the modern swine industry. However, the genome-wide mutational differences between these pig breeds are yet to be evaluated. Therefore, this study aimed to evaluate genomic variation and identify breed-specific SNPs in Danish LW pigs. Here, 43 LW, 15 Diqing Tibetan (DQZ), and 15 Diannan small-ear (DN) pigs whose genomes were re-sequenced with 5× depth were selected. This was followed by a conjoined analysis of our previous resequencing data of 24 Anqing six-end white (AQ) and six Asian wild (SS) pigs. In total, 39,158,378 SNPs and 13,143,989 insertion–deletions were obtained in all breeds. The variation number of LW pigs was the lowest, with 287,194 breed-specific and 1289 non-synonymous SNPs compared with Chinese breeds. Functional analysis of the breed-specific non-synonymous SNPs indicated that these mutations were mainly associated with the reproductive performance, feed intake, and feed conversion ratio of LW pigs. These findings provide a theoretical basis for genetic improvements in the Chinese swine industry.

## 1. Introduction

Genetic variation changes the genetic information of organisms. This affects phenotypic traits, which are not only the genetic basis of evolution and environmental adaptation but also the main source of biodiversity [1]. The rapid development of next-generation sequencing in recent years has provided an important technology for acquiring extensive genetic information. Whole-genome resequencing has been widely used in swine genomic research. For example, it is used for mutation site comparisons and analyses, such as SNP, insertion–deletion (InDel), and structural variation, based on the generation sequencing platform to re-sequence representative individuals of different groups within a species containing a known genome [2]. Understanding genetic variability and its influence on economically important traits is critical for pig breeding and production. High-quality, richly annotated reference genome sequences are key resources. They provide an important framework for discovering and analyzing genetic variation and linking genotypes to function. The new assembly of a Duroc pig (*Sus scrofa* 11.1) whole-genome sequence has provided a good reference [3].

Son et al. (2020) re-sequenced 20 micro-pigs to detect 144,507 non-synonymous SNPs, revealing that *RHEB* and *FRAS1* may affect traits of interest in these pigs [4]. Whole-genome re-sequencing data revealed that the number of genomic variants was similar in Danish Duroc, Landrace, and Large White pigs (LW), with almost half of the variants segregated in all three breeds. Moreover, LW pigs had 3,883,826 variants that are not found in Duroc or Landrace pigs [5]. The European and Asian wild boar populations diverged approximately 1 mya, and different selective directions resulted in millions of different minor allele frequencies of genomic locations [6]. As one of the most famous commercial lean pig breeds, Large White pigs (LW) were originally bred in Yorkshire, Britain, and then widely distributed in European and American countries. They have been selectively bred and improved according to different directions, resulting in various characteristic strains such as the Danish LW, American LW, Canadian LW, and French LW. There are significant phenotypic differences between LW and Chinese native pig breeds, with several hundred years of history of evolution and breeding. Compared with traditional breeding methods, marker-assisted selection can accurately and quickly analyze the genetic composition of individuals from the molecular level to realize the selection of genotypes with more information and higher selection accuracy. In animal breeding, the use of marker genotypes can accurately estimate the breeding value of quantitative traits. Danish LW pigs have a higher feed conversion ratio, growth rate, and lean meat percentage than local Chinese pigs. Thus, the molecular markers of their germplasm characteristics warrant further study to enable genetic improvement and hybrid breeding in the Chinese swine industry. This study aimed to investigate specific SNPs and their functions in LW pigs using Chinese local pig genome data as a control.

## 2. Materials and Methods

In this study, all animal experimental procedures were performed according to the Guide for the Care and Use of Laboratory Animals (Ministry of Science and Technology of China, 2006). Genetic variation information of 103 pigs was detected. Of these, 43 ear tissues of LW pigs were collected from Huaibei City, Anhui province, People’s Republic of China. Fifteen blood samples each of Diqing Tibetan (DQT) and Diannan small-ear (DN) pigs were collected from the Diqing Tibetan Autonomous Prefecture and Xishuangbanna Dai Autonomous Prefecture, Yunnan province, China, respectively, and stored in a 2 mL anticoagulation tube at −20 °C. Genomic DNA was extracted from the ear and blood samples using the standard phenol–chloroform method [7]. The quality and concentration of the DNA were assessed using a 1% agarose gel (run for >25 min at 120 V) and a NanoDrop spectrophotometer (Thermo Fisher Scientific, Waltham, MA, USA). Genomic DNA was randomly fragmented, and the fragmented genomic DNA was selected to a certain average size. The fragments were subjected to end-repair and then were 3′ adenylated. Then, adaptors were ligated to the ends of these 3′ adenylated fragments. After adapter ligation and DNA cluster preparation, the fragments were sequenced on the DNB-SEQ platform with 5× depth (Beijing Genomics Institution, Wuhan, China). The whole-genome data of 30 pigs, including 24 Anqing six-end white (AQ) and 6 Asian *Sus scrofa* (SS) pigs, were obtained from our previous work [8].

The comparison software bwa 0.7.17 (http://bio-bwa.sourceforge.net/ (accessed on 28 December 2023)) was used to compare the data to the reference genome (*Sus scrofa* 11.1) and the comparison samtools software 1.17 (https://github.com/samtools/samtools (accessed on 28 December 2023)) was used to sort the results and mark PCR duplications. We used the GATK software 4.4.0 (https://gatk.broadinstitute.org (accessed on 28 December 2023)) to find SNP and Indel sites. The detected variation was filtered using VariantFiltration, and quality control standards were set as -Window 4, -filter “QD < 4.0 || FS > 60.0 || MQ < 40.0”, -G_filter “GQ < 20” (QD:Variant Confidence/Quality by Depth; FS:Phred-scaled *p*-value using Fisher’s exact test to detect strand bias; MQ:RMS Mapping Quality; GQ:Genotype Quality). Then, ANNOVAR software 2020-06-07 (https://annovar.openbioinformatics.org/ (accessed on 28 December 2023)) was used to annotate the detected SNP, Indel, and other genomic variations with external databases.

Breed-specific SNPs were detected as follows in LW pigs, which were compared with four local Chinese pig breed genomes: (i) SNP only in the LW pig population, (ii) SNP only in autosomes, and (iii) SNP allele frequency > 0.1. The encompassing gene was annotated using the annotation database by NCBI (https://www.ncbi.nlm.nih.gov (accessed on 23 November 2023)), and to further analyze the functions of the identified genes, Gene Ontology (GO) and Kyoto Encyclopedia of Genes and Genomes (KEGG) analyses were performed. Additionally, an extensive literature review was performed to gather pertinent information on gene functions for exploratory investigations.

## 3. Results

**Figure 1:**
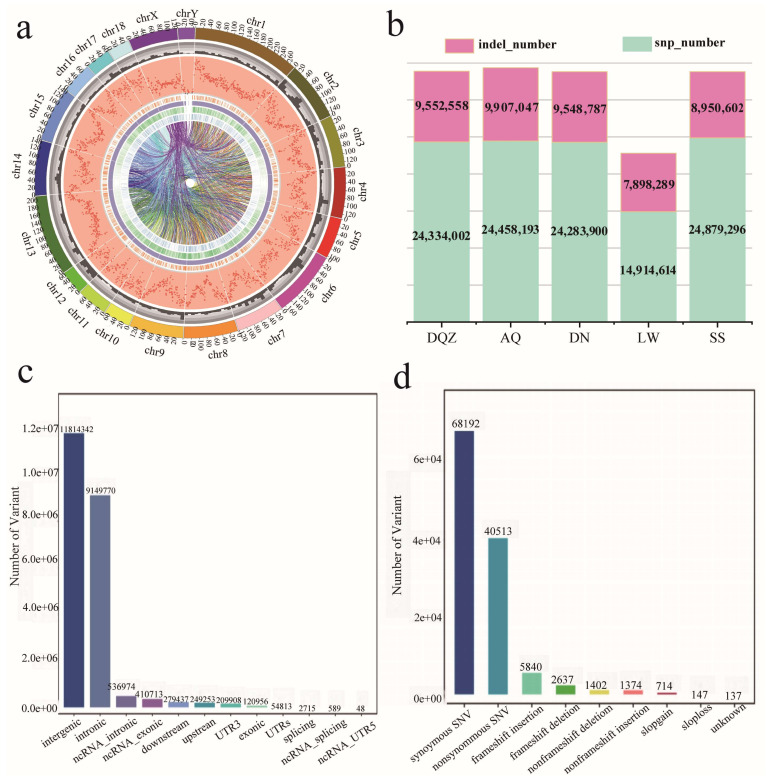
(**a**) The variation distribution in five pig breed genomes. Rings represent the genomic information of five pig breeds at each level. From the outside: length of each chromosome (in Mb). The orange, purple, green, and blue lines indicate the locations of tandem duplications (DUPs), large-segment deletions (DELs), insertion-type structural variations (INSs), and inverted-type structural variations (INVs) on the chromosome, respectively. The line in the inner circle indicates the position of BND (structural variant of the inter-chromosomal translocation type) on both chromosomes. (**b**) Number of single-nucleotide polymorphism (SNP) and insertion–deletion (InDel) statistics in five pig breed genomes. (**c**) Number of functional site statistics in the Large White (LW) pig genome. (**d**) Number of annotated site statistics in the LW pig genome.

In this study, 103 pigs from 5 pig populations were selected for whole-genome research, and approximately 10 billion reads (1406 Gb) were generated. Average data sets consisted of 13.65 Gb (5.46 X) each, and reads were aligned to the pig reference genome with an average alignment rate of 98.09 ± 0.02% and coverage (1×) with an average rate of 95.81 ± 0.40% (Supplementary Table S1). Of the 39,158,378 SNPs and 13,143,989 InDels detected in all animals after alignment with the reference genome 11.1 and variant calling, 14,914,614 SNPs and 7,898,289 InDels were detected in the LW pig genome, 24,334,002 SNPs and 9,552,558 InDels were detected in the DQZ pig genome, 24,458,193 SNPs and 9,907,047 InDels were detected in the AQ pig genome, 24,283,900 SNPs and 9,548,787 InDels were detected in the DN pig genome, and 24,879,296 SNPs and 8,950,602 InDels were detected in the SS pig genome (Figure 1a,b). Functional annotation results showed that 51.75%, 40.08%, and 5.30% of the SNPs in LW pigs were found in genome intergenic, intronic, and exonic regions, respectively (Figure 1c). Moreover, the ratio of exonic region SNPs to total SNPs in local Chinese pigs was higher than that in LW pigs, and the average transition/transversion (ts/tv) ratio of LW pigs was the lowest among the five pig breeds (Figure 1d).

**Figure 2:**
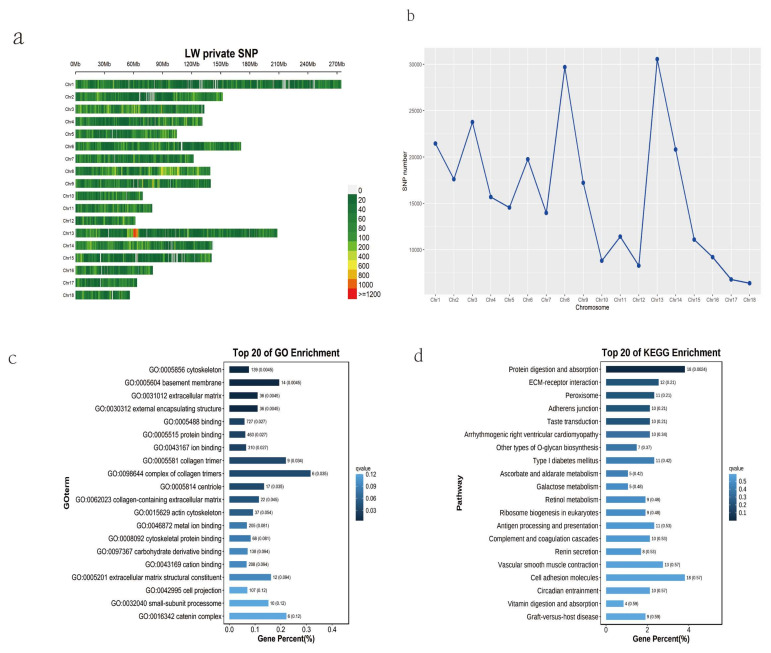
Breed-specific single-nucleotide polymorphisms (SNPs) and their functional analysis in LW pigs. (**a**) Distribution of breed-specific SNPs in the LW autosome. (**b**) Number statistics of breed-specific SNPs in the LW autosome. (**c**) Gene Ontology (GO) analysis of breed-specific non-synonymous SNPs. (**d**) Kyoto Encyclopedia of Genes and Genomes (KEGG) analysis of breed-specific non-synonymous SNPs.

We obtained 287,194 breed-specific SNPs in LW pigs, with *Sus scrofa* chromosomes (SSC) 13 and 18 showing the highest and lowest numbers of breed-specific SNPs, respectively (Figure 1a,b). The 2794 SNPs found in the exonic regions included 1486 synonymous, 1289 non-synonymous, 17 stop-gain, and 2 stoploss SNPs. We annotated 1131 genes of the LW pig breed-specific non-synonymous SNPs to further evaluate the functions of the identified genes using GO and KEGG analyses (Supplementary Table S2). These genes included 7575 GO items that were mainly enriched in the cytoskeleton, basement membrane, extracellular matrix, and external encapsulating structure terms (Figure 2c). KEGG pathway analysis revealed that the identified genes were enriched in protein digestion and absorption, ECM–receptor interaction, peroxisomes, adherens junction, and taste transduction pathways (Figure 2d). An extensive literature search revealed that *COL5A1*, *CSF1R*, *CEBPA*, *CYP4B1*, and *NQO1* were associated with back fat thickness and fat deposition; *COL27A1*, *PDZRN4*, *LTBP2*, *TBC1D1*, *CCSER1*, *KDM6B*, *GUCY2D*, *CPS1*, and *GCK* were associated with growth, body size, and carcass traits; *SPAG6*, *NUAK1*, *ZDHHC18*, and *ADAT1* were associated with reproductive performance; and *RAC1*, *AQP4*, *CYP2J2*, and *CDH19* were associated with feed intake and feed conversion ratio.

## 4. Discussion

Pigs are important domestic animals in China. The national pork production was 55.41 million tons in 2022, increasing by 4.6% year on year (https://www.stats.gov.cn/ (accessed on 28 December 2023)). In addition, pork consumption has always occupied a large proportion of the daily diet. Local pig breeds are abundant in China, with 83 native breeds (https://www.moa.gov.cn (accessed on 28 December 2023)) that exhibit good meat quality, reproductive performance, and disease resistance, providing valuable genetic resources. However, most local breeds have difficulty adapting to the modern swine industry because of their body size, lean percentages, and growth rates. Duroc, Landrace, and LW pigs are the main commercial breeds on large-scale pig farms in China. Therefore, the molecular marker identification of key traits and crossbreeding between lean and local breeds are important directions for pig production.

The genome is a complete collection of DNA, and whole-genome re-sequencing enables comprehensive genome-wide studies that elucidate all classes of genome variation across the allele frequency spectrum [9]. Many studies have revealed the breed characteristics of LW pigs using performance differences, the transcriptome, selective sweeps, and ROH analysis, identifying many candidate genes [10,11,12]. However, a genome-wide comparison of mutational differences between LW pigs and local Chinese pigs has not been performed. Major genomic and phenotypic differences have been observed between Asian and European pig breeds. However, the current reference genome belongs to the European Duroc pig, which is a lean-type breed similar to the LW pig. This explains why we obtained fewer variation loci in LW pigs than in local Chinese pigs. In the present study, most variations were in the intergenic and intronic regions of the genomes of all five pig breeds, which is consistent with previous reports. Breed-specific SNPs can elucidate the genomic landscape related to the unique characteristics of a breed compared with other breeds, which implies that they may be excellent genetic markers [13].

Non-synonymous SNPs in the protein-encoding regions of genes result in single amino acid substitutions in proteins, which causes considerable changes owing to the alteration in protein structure and function [14]. This may represent the genetic basis of characteristic differences in organisms. In the present study, 1131 identified genes possessing LW pig breed-specific non-synonymous SNPs were enriched in pathways related to transport, catabolism, metabolism, and digestion. Feed efficiency has a major ecological impact on pig production. Therefore, we focused on the candidate genes associated with the feed conversion ratio in this study. RACI proteins induce actin polymerization, which was upregulated in the high-feed-efficiency group of Duroc × (Landrace × Yorkshire) commercial pigs [15]. Endurance exercise increases *AQP4* expression levels in muscle cell membranes to regulate metabolic needs during physical activity. These expression levels have been linked to feed efficiency traits in GWAS results [16]. Vitamin A metabolism in the liver affects feed efficiency through energy metabolism and the promotion of fatty acid biosynthesis in pigs. *CYP2J2* is the key gene associated with fatty acid biosynthesis, and its expression is significantly upregulated in the livers of high-feed-efficiency pigs [17]. Similarly, *CDH19* plays an important role in the formation and maintenance of the integrity of various tissues. This gene has also been described on SSC1, with functions related to feed efficiency traits [18].

## 5. Conclusions

This study preliminarily revealed the genomic variation differences between LW and local Chinese pigs. We further described breed-specific genomic SNPs and their functions in LW pigs. Our results revealed 287,194 breed-specific genomic SNPs in LW pigs, which contained 1289 non-synonymous SNPs. Functional analysis of non-synonymous SNPs provides a reference for the genetic basis of LW pig characteristics, such as growth and feed conversion ratio. Finally, our findings may help develop useful molecular marker sites for application in the swine industry.

## Data Availability

The WGS datasets of LW, DQZ, and DN pigs obtained during the present study are not publicly available because of intellectual property considerations but are available from the corresponding author upon reasonable request. The WGS datasets of the AQ and SS pigs were submitted to the NCBI (National Center for Biotechnology Information, https://www.ncbi.nlm.nih.gov/ (accessed on 26 October 2023)) under accession number PRJNA699491.

## Supplementary Materials

The following supporting information can be downloaded at: https://www.mdpi.com/article/10.3390/genes15050623/s1, Table S1: The re-sequencing information of 103 samples. Table S2: The annotated genes information of the LW pig breed-specific non-synonymous SNPs.
