# Supplementary material for: Identification of Breed-Specific SNPs of Danish Large White Pig in Comparison with Four Chinese Local Pig Breed Genomes
Source: Genes (Basel). 2024 May 14;15(5):623. doi: 10.3390/genes15050623 (PMC11120843; doi:10.3390/genes15050623)
Supplement: Supplementary file 1 [file genes-15-00623-s001.zip › Supplementary Table S2 The annotated genes information of the LW pig breed-specific non-synonymous SNPs.pdf]

supplementary table S2 The annotated genes information of the LW pig breed-specific non-synonymous SNPs

#chr	start	end	structure_type	function_type	GeneID
1	513579	513579	exonic	nonsynonymous SNV	ENSSSCG00000004010
1	1584076	1584076	exonic	nonsynonymous SNV	ENSSSCG00000004018
1	1939671	1939671	exonic	nonsynonymous SNV	ENSSSCG00000027404
1	2204022	2204022	exonic	nonsynonymous SNV	ENSSSCG00000004022
1	7372173	7372173	exonic	nonsynonymous SNV	ENSSSCG00000004044
1	9811295	9811295	exonic	nonsynonymous SNV	ENSSSCG00000004064
1	14046203	14046203	exonic	nonsynonymous SNV	ENSSSCG00000004082
1	14659837	14659837	exonic	nonsynonymous SNV	ENSSSCG00000004087
1	16025403	16025403	exonic	nonsynonymous SNV	ENSSSCG00000032525
1	17314322	17314322	exonic	nonsynonymous SNV	ENSSSCG00000033175
1	19765552	19765552	exonic	nonsynonymous SNV	ENSSSCG00000004121
1	25980052	25980052	exonic	nonsynonymous SNV	ENSSSCG00000004149
1	27051817	27051817	exonic	nonsynonymous SNV	ENSSSCG00000004156
1	27088970	27088970	exonic	nonsynonymous SNV	ENSSSCG00000004158
1	27769438	27769438	exonic	nonsynonymous SNV	ENSSSCG00000039049
1	27918948	27918948	exonic	nonsynonymous SNV	ENSSSCG00000004164
1	30988145	30988145	exonic	nonsynonymous SNV	ENSSSCG00000004180
1	33815915	33815915	exonic	nonsynonymous SNV	ENSSSCG00000004207
1	34074109	34074109	exonic	nonsynonymous SNV	ENSSSCG00000042942
1	35766902	35766902	exonic	nonsynonymous SNV	ENSSSCG00000004215
1	45268688	45268688	exonic	nonsynonymous SNV	ENSSSCG00000035259
1	45293307	45293307	exonic	nonsynonymous SNV	ENSSSCG00000029003
1	56689242	56689242	exonic	nonsynonymous SNV	ENSSSCG00000004342
1	64044670	64044670	exonic	nonsynonymous SNV	ENSSSCG00000037612
1	71295145	71295145	exonic	nonsynonymous SNV	ENSSSCG00000004412
1	75516347	75516347	exonic	nonsynonymous SNV	ENSSSCG00000004405
1	75791406	75791406	exonic	nonsynonymous SNV	ENSSSCG00000004460
1	84009167	84009167	exonic	nonsynonymous SNV	ENSSSCG00000004521
1	100345971	100345971	exonic	nonsynonymous SNV	ENSSSCG00000004823
1	139542207	139542207	exonic	nonsynonymous SNV	ENSSSCG00000004847
1	143980510	143980510	exonic	nonsynonymous SNV	ENSSSCG00000004885
1	155727821	155727821	exonic	nonsynonymous SNV	ENSSSCG00000004929
1	163303116	163303116	exonic	nonsynonymous SNV	ENSSSCG00000004941
1	164337095	164337095	exonic	nonsynonymous SNV	ENSSSCG00000004957
1	166125890	166125890	exonic	nonsynonymous SNV	ENSSSCG00000004973
1	168390459	168390459	exonic	nonsynonymous SNV	ENSSSCG00000061989
1	174732987	174732987	exonic	nonsynonymous SNV	ENSSSCG00000004999
1	174733451	174733451	exonic	nonsynonymous SNV	ENSSSCG00000005004
1	175404585	175404585	exonic	nonsynonymous SNV	ENSSSCG00000005068
1	179469474	179469474	exonic	nonsynonymous SNV	ENSSSCG00000032928
1	186713033	186713033	exonic	nonsynonymous SNV	ENSSSCG00000005081
1	188581636	188581636	exonic	nonsynonymous SNV	ENSSSCG00000005101

1	188582115	188582115	exonic	nonsynonymous SNV	ENSSSCG00000060359
1	189017515	189017515	exonic	nonsynonymous SNV	ENSSSCG00000033910
1	191102327	191102327	exonic	nonsynonymous SNV	ENSSSCG00000028406
1	201342852	201342852	exonic	nonsynonymous SNV	ENSSSCG00000005169
1	201623993	201623993	exonic	nonsynonymous SNV	ENSSSCG00000005170
1	202026738	202026738	exonic	nonsynonymous SNV	ENSSSCG00000005282
1	203446086	203446086	exonic	nonsynonymous SNV	ENSSSCG00000037497
1	203484898	203484898	exonic	nonsynonymous SNV	ENSSSCG00000005305
1	230214368	230214368	exonic	nonsynonymous SNV	ENSSSCG00000005313
1	234580261	234580261	exonic	nonsynonymous SNV	ENSSSCG00000048089
1	236051469	236051469	exonic	nonsynonymous SNV	ENSSSCG00000005350
1	236383330	236383330	exonic	nonsynonymous SNV	ENSSSCG00000005449
1	236728434	236728434	exonic	nonsynonymous SNV	ENSSSCG00000005459
1	238251083	238251083	exonic	nonsynonymous SNV	ENSSSCG00000020987
1	250462750	250462750	exonic	nonsynonymous SNV	ENSSSCG00000005487
1	252231066	252231066	exonic	nonsynonymous SNV	ENSSSCG00000005494
1	253953545	253953545	exonic	nonsynonymous SNV	ENSSSCG00000005524
1	254849366	254849366	exonic	nonsynonymous SNV	ENSSSCG00000031440
1	255596979	255596979	exonic	nonsynonymous SNV	ENSSSCG00000031426
1	261756783	261756783	exonic	nonsynonymous SNV	ENSSSCG00000005625
1	266381998	266381998	exonic	nonsynonymous SNV	ENSSSCG00000005623
1	267460116	267460116	exonic	nonsynonymous SNV	ENSSSCG00000005628
1	268315026	268315026	exonic	nonsynonymous SNV	ENSSSCG00000005636
1	268382480	268382480	exonic	nonsynonymous SNV	ENSSSCG00000005652
1	268392391	268392391	exonic	nonsynonymous SNV	ENSSSCG00000063201
1	268561034	268561034	exonic	nonsynonymous SNV	ENSSSCG00000005669
1	268906624	268906624	exonic	nonsynonymous SNV	ENSSSCG00000005671
1	269084784	269084784	exonic	nonsynonymous SNV	ENSSSCG00000005672
1	269338162	269338162	exonic	nonsynonymous SNV	ENSSSCG00000005711
1	269391399	269391399	exonic	nonsynonymous SNV	ENSSSCG00000035964
1	269419092	269419092	exonic	nonsynonymous SNV	ENSSSCG00000005751
1	271173606	271173606	exonic	nonsynonymous SNV	ENSSSCG00000014559
1	272890445	272890445	exonic	nonsynonymous SNV	ENSSSCG00000032010
1	274082798	274082798	exonic	nonsynonymous SNV	ENSSSCG00000012835
2	71478	71478	exonic	nonsynonymous SNV	ENSSSCG00000033382
2	283624	283624	exonic	nonsynonymous SNV	ENSSSCG00000032911
2	649282	649282	exonic	nonsynonymous SNV	ENSSSCG00000023537
2	762699	762699	exonic	nonsynonymous SNV	ENSSSCG00000036244
2	763773	763773	exonic	nonsynonymous SNV	ENSSSCG00000029902
2	764150	764150	exonic	nonsynonymous SNV	ENSSSCG00000038508
2	765196	765196	exonic	nonsynonymous SNV	ENSSSCG00000021891
2	1015139	1015139	exonic	nonsynonymous SNV	ENSSSCG00000012959
2	1248654	1248654	exonic	nonsynonymous SNV	ENSSSCG00000012992
2	1292950	1292950	exonic	nonsynonymous SNV	ENSSSCG00000039740

2	5623657	5623657	exonic	nonsynonymous SNV	ENSSSCG00000013025
2	5719936	5719936	exonic	nonsynonymous SNV	ENSSSCG00000013056
2	5720404	5720404	exonic	nonsynonymous SNV	ENSSSCG00000028025
2	5881323	5881323	exonic	nonsynonymous SNV	ENSSSCG00000022866
2	6313235	6313235	exonic	nonsynonymous SNV	ENSSSCG00000055375
2	6827155	6827155	exonic	nonsynonymous SNV	ENSSSCG00000021906
2	7063052	7063052	exonic	nonsynonymous SNV	ENSSSCG00000058512
2	7063976	7063976	exonic	nonsynonymous SNV	ENSSSCG00000013090
2	7561368	7561368	exonic	nonsynonymous SNV	ENSSSCG00000026060
2	8472694	8472694	exonic	nonsynonymous SNV	ENSSSCG00000062785
2	8933990	8933990	exonic	nonsynonymous SNV	ENSSSCG00000013098
2	8975511	8975511	exonic	nonsynonymous SNV	ENSSSCG00000013111
2	9206796	9206796	exonic	nonsynonymous SNV	ENSSSCG00000022114
2	9206797	9206797	exonic	nonsynonymous SNV	ENSSSCG00000013102
2	9983789	9983789	exonic	nonsynonymous SNV	ENSSSCG00000013119
2	10342895	10342895	exonic	nonsynonymous SNV	ENSSSCG00000013174
2	10342910	10342910	exonic	nonsynonymous SNV	ENSSSCG00000031326
2	10431337	10431337	exonic	nonsynonymous SNV	ENSSSCG00000052696
2	10433102	10433102	exonic	nonsynonymous SNV	ENSSSCG00000054225
2	10448367	10448367	exonic	nonsynonymous SNV	ENSSSCG00000013231
2	10542472	10542472	exonic	nonsynonymous SNV	ENSSSCG00000013239
2	10542474	10542474	exonic	nonsynonymous SNV	ENSSSCG00000013245
2	10596783	10596783	exonic	nonsynonymous SNV	ENSSSCG00000013252
2	10752002	10752002	exonic	nonsynonymous SNV	ENSSSCG00000028338
2	11271143	11271143	exonic	nonsynonymous SNV	ENSSSCG00000013297
2	11310092	11310092	exonic	nonsynonymous SNV	ENSSSCG00000013301
2	11740637	11740637	exonic	nonsynonymous SNV	ENSSSCG00000013302
2	13159464	13159464	exonic	nonsynonymous SNV	ENSSSCG00000013318
2	13545351	13545351	exonic	nonsynonymous SNV	ENSSSCG00000013326
2	14216016	14216016	exonic	nonsynonymous SNV	ENSSSCG00000013377
2	14444951	14444951	exonic	nonsynonymous SNV	ENSSSCG00000031706
2	15025962	15025962	exonic	nonsynonymous SNV	ENSSSCG00000038176
2	15180606	15180606	exonic	nonsynonymous SNV	ENSSSCG00000034569
2	15397558	15397558	exonic	nonsynonymous SNV	ENSSSCG00000044276
2	15813707	15813707	exonic	nonsynonymous SNV	ENSSSCG00000035155
2	25590589	25590589	exonic	nonsynonymous SNV	ENSSSCG00000055945
2	25825459	25825459	exonic	nonsynonymous SNV	ENSSSCG00000056866
2	26464759	26464759	exonic	nonsynonymous SNV	ENSSSCG00000013941
2	26496799	26496799	exonic	nonsynonymous SNV	ENSSSCG00000053785
2	28709296	28709296	exonic	nonsynonymous SNV	ENSSSCG00000013895
2	29721336	29721336	exonic	nonsynonymous SNV	ENSSSCG00000041467
2	29755069	29755069	exonic	nonsynonymous SNV	ENSSSCG00000027013
2	41621454	41621454	exonic	nonsynonymous SNV	ENSSSCG00000026780
2	41640439	41640439	exonic	nonsynonymous SNV	ENSSSCG00000014163

2	47139985	47139985	exonic	nonsynonymous SNV	ENSSSCG00000026175
2	50070281	50070281	exonic	nonsynonymous SNV	ENSSSCG00000014140
2	51324672	51324672	exonic	nonsynonymous SNV	ENSSSCG00000014168
2	51524050	51524050	exonic	nonsynonymous SNV	ENSSSCG00000014173
2	53209726	53209726	exonic	nonsynonymous SNV	ENSSSCG00000014179
2	53594845	53594845	exonic	nonsynonymous SNV	ENSSSCG00000014203
2	54215116	54215116	exonic	nonsynonymous SNV	ENSSSCG00000022048
2	55135176	55135176	exonic	nonsynonymous SNV	ENSSSCG00000014207
2	56862319	56862319	exonic	nonsynonymous SNV	ENSSSCG00000022402
2	57689721	57689721	exonic	nonsynonymous SNV	ENSSSCG00000014247
2	59655034	59655034	exonic	nonsynonymous SNV	ENSSSCG00000014258
2	60314962	60314962	exonic	nonsynonymous SNV	ENSSSCG00000038953
2	61904610	61904610	exonic	nonsynonymous SNV	ENSSSCG00000014303
2	92126807	92126807	exonic	nonsynonymous SNV	ENSSSCG00000026106
2	101368612	101368612	exonic	nonsynonymous SNV	ENSSSCG00000023691
2	102014302	102014302	exonic	nonsynonymous SNV	ENSSSCG00000014335
2	102223248	102223248	exonic	nonsynonymous SNV	ENSSSCG00000014339
2	102552442	102552442	exonic	nonsynonymous SNV	ENSSSCG00000014362
2	103604579	103604579	exonic	nonsynonymous SNV	ENSSSCG00000029281
2	103605668	103605668	exonic	nonsynonymous SNV	ENSSSCG00000027371
2	106555639	106555639	exonic	nonsynonymous SNV	ENSSSCG00000056350
2	116718140	116718140	exonic	nonsynonymous SNV	ENSSSCG00000014410
2	116846708	116846708	exonic	nonsynonymous SNV	ENSSSCG00000014408
2	116878963	116878963	exonic	nonsynonymous SNV	ENSSSCG00000014415
2	116918379	116918379	exonic	nonsynonymous SNV	ENSSSCG00000039045
2	129590514	129590514	exonic	nonsynonymous SNV	ENSSSCG00000014440
2	129706595	129706595	exonic	nonsynonymous SNV	ENSSSCG00000014441
2	131867531	131867531	exonic	nonsynonymous SNV	ENSSSCG00000014450
2	136661695	136661695	exonic	nonsynonymous SNV	ENSSSCG00000032731
2	136827593	136827593	exonic	nonsynonymous SNV	ENSSSCG00000021427
2	137078685	137078685	exonic	nonsynonymous SNV	ENSSSCG00000056671
2	140328000	140328000	exonic	nonsynonymous SNV	ENSSSCG00000007563
2	140425725	140425725	exonic	nonsynonymous SNV	ENSSSCG00000007577
2	140682816	140682816	exonic	nonsynonymous SNV	ENSSSCG00000007575
2	142114567	142114567	exonic	nonsynonymous SNV	ENSSSCG00000022553
2	142578901	142578901	exonic	nonsynonymous SNV	ENSSSCG00000008665
2	143132761	143132761	exonic	nonsynonymous SNV	ENSSSCG00000037070
2	143136656	143136656	exonic	nonsynonymous SNV	ENSSSCG00000027288
2	144463018	144463018	exonic	nonsynonymous SNV	ENSSSCG00000035090
2	147350505	147350505	exonic	nonsynonymous SNV	ENSSSCG00000032705
2	147453830	147453830	exonic	nonsynonymous SNV	ENSSSCG00000007599
2	147856747	147856747	exonic	nonsynonymous SNV	ENSSSCG00000007602
2	151018088	151018088	exonic	nonsynonymous SNV	ENSSSCG00000035534
2	151018499	151018499	exonic	nonsynonymous SNV	ENSSSCG00000007622

2	151045111	151045111	exonic	nonsynonymous SNV	ENSSSCG00000036665
2	151115268	151115268	exonic	nonsynonymous SNV	ENSSSCG00000029175
2	151379870	151379870	exonic	nonsynonymous SNV	ENSSSCG00000038518
2	151489911	151489911	exonic	nonsynonymous SNV	ENSSSCG00000039119
2	151762627	151762627	exonic	nonsynonymous SNV	ENSSSCG00000060711
2	151762627	151762627	exonic	nonsynonymous SNV	ENSSSCG00000032180
3	1912979	1912979	exonic	nonsynonymous SNV	ENSSSCG00000007644
3	1913030	1913030	exonic	nonsynonymous SNV	ENSSSCG00000007650
3	1917928	1917928	exonic	nonsynonymous SNV	ENSSSCG00000007659
3	3634208	3634208	exonic	nonsynonymous SNV	ENSSSCG00000030337
3	3648983	3648983	exonic	nonsynonymous SNV	ENSSSCG00000028879
3	3907467	3907467	exonic	nonsynonymous SNV	ENSSSCG00000007681
3	4516956	4516956	exonic	nonsynonymous SNV	ENSSSCG00000007703
3	4517199	4517199	exonic	nonsynonymous SNV	ENSSSCG00000007716
3	4683426	4683426	exonic	nonsynonymous SNV	ENSSSCG00000007751
3	4744843	4744843	exonic	nonsynonymous SNV	ENSSSCG00000007780
3	4783513	4783513	exonic	nonsynonymous SNV	ENSSSCG00000007789
3	5009142	5009142	exonic	nonsynonymous SNV	ENSSSCG00000007801
3	5379546	5379546	exonic	nonsynonymous SNV	ENSSSCG00000027946
3	5395377	5395377	exonic	nonsynonymous SNV	ENSSSCG00000026748
3	5395866	5395866	exonic	nonsynonymous SNV	ENSSSCG00000010794
3	5396215	5396215	exonic	nonsynonymous SNV	ENSSSCG00000007838
3	5396245	5396245	exonic	nonsynonymous SNV	ENSSSCG00000030373
3	5396401	5396401	exonic	nonsynonymous SNV	ENSSSCG00000035000
3	5396434	5396434	exonic	nonsynonymous SNV	ENSSSCG00000007847
3	5488639	5488639	exonic	nonsynonymous SNV	ENSSSCG00000046747
3	6233587	6233587	exonic	nonsynonymous SNV	ENSSSCG00000007857
3	6237037	6237037	exonic	nonsynonymous SNV	ENSSSCG00000007866
3	6365296	6365296	exonic	nonsynonymous SNV	ENSSSCG00000040412
3	6431242	6431242	exonic	nonsynonymous SNV	ENSSSCG00000032381
3	6494748	6494748	exonic	nonsynonymous SNV	ENSSSCG00000024307
3	6794672	6794672	exonic	nonsynonymous SNV	ENSSSCG00000033311
3	7514842	7514842	exonic	nonsynonymous SNV	ENSSSCG00000007914
3	7679636	7679636	exonic	nonsynonymous SNV	ENSSSCG00000043838
3	8006070	8006070	exonic	nonsynonymous SNV	ENSSSCG00000021569
3	8100148	8100148	exonic	nonsynonymous SNV	ENSSSCG00000030518
3	8195353	8195353	exonic	nonsynonymous SNV	ENSSSCG00000008055
3	8195383	8195383	exonic	nonsynonymous SNV	ENSSSCG00000008051
3	8336237	8336237	exonic	nonsynonymous SNV	ENSSSCG00000052991
3	8347210	8347210	exonic	nonsynonymous SNV	ENSSSCG00000008047
3	8415088	8415088	exonic	nonsynonymous SNV	ENSSSCG00000008041
3	8915076	8915076	exonic	nonsynonymous SNV	ENSSSCG00000008013
3	9661194	9661194	exonic	nonsynonymous SNV	ENSSSCG00000021467
3	10647921	10647921	exonic	nonsynonymous SNV	ENSSSCG00000008020

3	11078183	11078183	exonic	nonsynonymous SNV	ENSSSCG00000048138
3	17002533	17002533	exonic	nonsynonymous SNV	ENSSSCG00000034266
3	17615470	17615470	exonic	nonsynonymous SNV	ENSSSCG00000008010
3	17665071	17665071	exonic	nonsynonymous SNV	ENSSSCG00000008002
3	17666468	17666468	exonic	nonsynonymous SNV	ENSSSCG00000058140
3	17925221	17925221	exonic	nonsynonymous SNV	ENSSSCG00000007991
3	18081104	18081104	exonic	nonsynonymous SNV	ENSSSCG00000008068
3	22540505	22540505	exonic	nonsynonymous SNV	ENSSSCG00000034154
3	22594571	22594571	exonic	nonsynonymous SNV	ENSSSCG00000008122
3	23645193	23645193	exonic	nonsynonymous SNV	ENSSSCG00000031072
3	23720245	23720245	exonic	nonsynonymous SNV	ENSSSCG00000008135
3	24320931	24320931	exonic	nonsynonymous SNV	ENSSSCG00000008138
3	24572953	24572953	exonic	nonsynonymous SNV	ENSSSCG00000008155
3	24960117	24960117	exonic	nonsynonymous SNV	ENSSSCG00000008165
3	25262463	25262463	exonic	nonsynonymous SNV	ENSSSCG00000008169
3	26323416	26323416	exonic	nonsynonymous SNV	ENSSSCG00000008171
3	28816532	28816532	exonic	nonsynonymous SNV	ENSSSCG00000008194
3	31574798	31574798	exonic	nonsynonymous SNV	ENSSSCG00000008197
3	31864514	31864514	exonic	nonsynonymous SNV	ENSSSCG00000025106
3	33894170	33894170	exonic	nonsynonymous SNV	ENSSSCG00000008213
3	34049173	34049173	exonic	nonsynonymous SNV	ENSSSCG00000023710
3	38936146	38936146	exonic	nonsynonymous SNV	ENSSSCG00000008239
3	39133702	39133702	exonic	nonsynonymous SNV	ENSSSCG00000008241
3	39303452	39303452	exonic	nonsynonymous SNV	ENSSSCG00000045529
3	39580662	39580662	exonic	nonsynonymous SNV	ENSSSCG00000027320
3	39708146	39708146	exonic	nonsynonymous SNV	ENSSSCG00000008292
3	39755302	39755302	exonic	nonsynonymous SNV	ENSSSCG00000023258
3	39762852	39762852	exonic	nonsynonymous SNV	ENSSSCG00000008344
3	39865589	39865589	exonic	nonsynonymous SNV	ENSSSCG00000023908
3	39897597	39897597	exonic	nonsynonymous SNV	ENSSSCG00000026559
3	39898136	39898136	exonic	nonsynonymous SNV	ENSSSCG00000029084
3	40177350	40177350	exonic	nonsynonymous SNV	ENSSSCG00000008467
3	40191363	40191363	exonic	nonsynonymous SNV	ENSSSCG00000008512
3	40364314	40364314	exonic	nonsynonymous SNV	ENSSSCG00000024781
3	40430601	40430601	exonic	nonsynonymous SNV	ENSSSCG00000037201
3	40647786	40647786	exonic	nonsynonymous SNV	ENSSSCG00000063152
3	41000908	41000908	exonic	nonsynonymous SNV	ENSSSCG00000043825
3	41049605	41049605	exonic	nonsynonymous SNV	ENSSSCG00000033511
3	41062390	41062390	exonic	nonsynonymous SNV	ENSSSCG00000008589
3	41107758	41107758	exonic	nonsynonymous SNV	ENSSSCG00000008595
3	41964577	41964577	exonic	nonsynonymous SNV	ENSSSCG00000040752
3	42777605	42777605	exonic	nonsynonymous SNV	ENSSSCG00000008621
3	42808173	42808173	exonic	nonsynonymous SNV	ENSSSCG00000025483
3	45761804	45761804	exonic	nonsynonymous SNV	ENSSSCG00000008649

3	46117672	46117672	exonic	nonsynonymous SNV	ENSSSCG00000006956
3	47516120	47516120	exonic	nonsynonymous SNV	ENSSSCG00000034820
3	47725432	47725432	exonic	nonsynonymous SNV	ENSSSCG00000039881
3	47751157	47751157	exonic	nonsynonymous SNV	ENSSSCG00000033726
3	51693006	51693006	exonic	nonsynonymous SNV	ENSSSCG00000005938
3	52889909	52889909	exonic	nonsynonymous SNV	ENSSSCG00000005939
3	53252694	53252694	exonic	nonsynonymous SNV	ENSSSCG00000005948
3	53300170	53300170	exonic	nonsynonymous SNV	ENSSSCG00000005983
3	56612640	56612640	exonic	nonsynonymous SNV	ENSSSCG00000005995
3	56757572	56757572	exonic	nonsynonymous SNV	ENSSSCG00000005997
3	57818814	57818814	exonic	nonsynonymous SNV	ENSSSCG00000006011
3	57971640	57971640	exonic	nonsynonymous SNV	ENSSSCG00000006037
3	57971807	57971807	exonic	nonsynonymous SNV	ENSSSCG00000044983
3	58536976	58536976	exonic	nonsynonymous SNV	ENSSSCG00000050468
3	58536984	58536984	exonic	nonsynonymous SNV	ENSSSCG00000024290
3	59357470	59357470	exonic	nonsynonymous SNV	ENSSSCG00000033791
3	59357502	59357502	exonic	nonsynonymous SNV	ENSSSCG00000048309
3	59458920	59458920	exonic	nonsynonymous SNV	ENSSSCG00000006185
3	59912694	59912694	exonic	nonsynonymous SNV	ENSSSCG00000006187
3	59996211	59996211	exonic	nonsynonymous SNV	ENSSSCG00000006206
3	68791493	68791493	exonic	nonsynonymous SNV	ENSSSCG00000034495
3	68925769	68925769	exonic	nonsynonymous SNV	ENSSSCG00000006274
3	71688611	71688611	exonic	nonsynonymous SNV	ENSSSCG00000006284
3	71688869	71688869	exonic	nonsynonymous SNV	ENSSSCG00000006305
3	73558309	73558309	exonic	nonsynonymous SNV	ENSSSCG00000006372
3	74724318	74724318	exonic	nonsynonymous SNV	ENSSSCG00000062362
3	86261615	86261615	exonic	nonsynonymous SNV	ENSSSCG00000006418
3	91774450	91774450	exonic	nonsynonymous SNV	ENSSSCG00000006431
3	98034098	98034098	exonic	nonsynonymous SNV	ENSSSCG00000053741
3	106829002	106829002	exonic	nonsynonymous SNV	ENSSSCG00000022686
3	108166772	108166772	exonic	nonsynonymous SNV	ENSSSCG00000030145
3	111821708	111821708	exonic	nonsynonymous SNV	ENSSSCG00000058723
3	111835205	111835205	exonic	nonsynonymous SNV	ENSSSCG00000006468
3	111855735	111855735	exonic	nonsynonymous SNV	ENSSSCG00000006470
3	112156094	112156094	exonic	nonsynonymous SNV	ENSSSCG00000006477
3	114642653	114642653	exonic	nonsynonymous SNV	ENSSSCG00000006495
3	117273113	117273113	exonic	nonsynonymous SNV	ENSSSCG00000006510
3	117276590	117276590	exonic	nonsynonymous SNV	ENSSSCG00000006518
3	117304683	117304683	exonic	nonsynonymous SNV	ENSSSCG00000006523
3	117306996	117306996	exonic	nonsynonymous SNV	ENSSSCG00000006533
3	117307292	117307292	exonic	nonsynonymous SNV	ENSSSCG00000028606
3	117311373	117311373	exonic	nonsynonymous SNV	ENSSSCG00000006546
3	117311945	117311945	exonic	nonsynonymous SNV	ENSSSCG00000006555
3	120021452	120021452	exonic	nonsynonymous SNV	ENSSSCG00000038991

3	121882891	121882891	exonic	nonsynonymous SNV	ENSSSCG00000053832
3	125117504	125117504	exonic	nonsynonymous SNV	ENSSSCG00000006604
3	125153500	125153500	exonic	nonsynonymous SNV	ENSSSCG00000026259
3	129799051	129799051	exonic	nonsynonymous SNV	ENSSSCG00000006619
4	1012822	1012822	exonic	nonsynonymous SNV	ENSSSCG00000022784
4	1092781	1092781	exonic	nonsynonymous SNV	ENSSSCG00000024481
4	1217758	1217758	exonic	nonsynonymous SNV	ENSSSCG00000006713
4	1551222	1551222	exonic	nonsynonymous SNV	ENSSSCG00000006720
4	4270802	4270802	exonic	nonsynonymous SNV	ENSSSCG00000006733
4	4640938	4640938	exonic	nonsynonymous SNV	ENSSSCG00000006746
4	8306129	8306129	exonic	nonsynonymous SNV	ENSSSCG00000032417
4	8322775	8322775	exonic	nonsynonymous SNV	ENSSSCG00000006755
4	16033374	16033374	exonic	nonsynonymous SNV	ENSSSCG00000006758
4	18571093	18571093	exonic	nonsynonymous SNV	ENSSSCG00000006801
4	18887111	18887111	exonic	nonsynonymous SNV	ENSSSCG00000006842
4	21524527	21524527	exonic	nonsynonymous SNV	ENSSSCG00000036509
4	30796035	30796035	exonic	nonsynonymous SNV	ENSSSCG00000006904
4	31266048	31266048	exonic	nonsynonymous SNV	ENSSSCG00000030801
4	35326376	35326376	exonic	nonsynonymous SNV	ENSSSCG00000062656
4	42344935	42344935	exonic	nonsynonymous SNV	ENSSSCG00000059930
4	54949027	54949027	exonic	nonsynonymous SNV	ENSSSCG00000031738
4	57607370	57607370	exonic	nonsynonymous SNV	ENSSSCG00000006939
4	62880473	62880473	exonic	nonsynonymous SNV	ENSSSCG00000000960
4	63710279	63710279	exonic	nonsynonymous SNV	ENSSSCG00000000964
4	67940314	67940314	exonic	nonsynonymous SNV	ENSSSCG00000023749
4	78633273	78633273	exonic	nonsynonymous SNV	ENSSSCG00000000981
4	79828333	79828333	exonic	nonsynonymous SNV	ENSSSCG00000031361
4	81207309	81207309	exonic	nonsynonymous SNV	ENSSSCG00000000002
4	83157093	83157093	exonic	nonsynonymous SNV	ENSSSCG00000024718
4	89375871	89375871	exonic	nonsynonymous SNV	ENSSSCG00000000014
4	89504404	89504404	exonic	nonsynonymous SNV	ENSSSCG00000000022
4	91391331	91391331	exonic	nonsynonymous SNV	ENSSSCG00000000033
4	91497439	91497439	exonic	nonsynonymous SNV	ENSSSCG00000000132
4	91712802	91712802	exonic	nonsynonymous SNV	ENSSSCG00000024881
4	91967738	91967738	exonic	nonsynonymous SNV	ENSSSCG00000032861
4	91980448	91980448	exonic	nonsynonymous SNV	ENSSSCG00000000195
4	92099909	92099909	exonic	nonsynonymous SNV	ENSSSCG00000032269
4	93340991	93340991	exonic	nonsynonymous SNV	ENSSSCG00000026061
4	93359070	93359070	exonic	nonsynonymous SNV	ENSSSCG00000028516
4	93432710	93432710	exonic	nonsynonymous SNV	ENSSSCG00000000232
4	93868019	93868019	exonic	nonsynonymous SNV	ENSSSCG00000000234
4	94246922	94246922	exonic	nonsynonymous SNV	ENSSSCG00000000235
4	94557720	94557720	exonic	nonsynonymous SNV	ENSSSCG00000023254
4	94609858	94609858	exonic	nonsynonymous SNV	ENSSSCG00000027548

4	94746630	94746630	exonic	nonsynonymous SNV	ENSSSCG00000000259
4	94783704	94783704	exonic	nonsynonymous SNV	ENSSSCG00000000265
4	95277410	95277410	exonic	nonsynonymous SNV	ENSSSCG00000000278
4	95534309	95534309	exonic	nonsynonymous SNV	ENSSSCG00000054230
4	96013216	96013216	exonic	nonsynonymous SNV	ENSSSCG00000000385
4	96073628	96073628	exonic	nonsynonymous SNV	ENSSSCG00000037617
4	96937852	96937852	exonic	nonsynonymous SNV	ENSSSCG00000037767
4	97404056	97404056	exonic	nonsynonymous SNV	ENSSSCG00000029571
4	97620533	97620533	exonic	nonsynonymous SNV	ENSSSCG00000000504
4	98925035	98925035	exonic	nonsynonymous SNV	ENSSSCG00000000515
4	100685376	100685376	exonic	nonsynonymous SNV	ENSSSCG00000000588
4	100729539	100729539	exonic	nonsynonymous SNV	ENSSSCG00000057970
4	101315546	101315546	exonic	nonsynonymous SNV	ENSSSCG00000000612
4	101594722	101594722	exonic	nonsynonymous SNV	ENSSSCG00000000619
4	103651592	103651592	exonic	nonsynonymous SNV	ENSSSCG00000044781
4	103662298	103662298	exonic	nonsynonymous SNV	ENSSSCG00000000634
4	104998771	104998771	exonic	nonsynonymous SNV	ENSSSCG00000040465
4	105307519	105307519	exonic	nonsynonymous SNV	ENSSSCG00000000642
4	105949504	105949504	exonic	nonsynonymous SNV	ENSSSCG00000024402
4	106439388	106439388	exonic	nonsynonymous SNV	ENSSSCG00000000668
4	109347381	109347381	exonic	nonsynonymous SNV	ENSSSCG00000025252
4	111195959	111195959	exonic	nonsynonymous SNV	ENSSSCG00000033913
4	122188610	122188610	exonic	nonsynonymous SNV	ENSSSCG00000058867
4	122190831	122190831	exonic	nonsynonymous SNV	ENSSSCG00000000728
4	124810267	124810267	exonic	nonsynonymous SNV	ENSSSCG00000029464
4	127238768	127238768	exonic	nonsynonymous SNV	ENSSSCG00000033412
4	127293724	127293724	exonic	nonsynonymous SNV	ENSSSCG00000026537
4	129492746	129492746	exonic	nonsynonymous SNV	ENSSSCG00000059113
4	129965575	129965575	exonic	nonsynonymous SNV	ENSSSCG00000041764
4	130158664	130158664	exonic	nonsynonymous SNV	ENSSSCG00000000779
4	130298827	130298827	exonic	nonsynonymous SNV	ENSSSCG00000000781
5	203083	203083	exonic	nonsynonymous SNV	ENSSSCG00000000791
5	235036	235036	exonic	nonsynonymous SNV	ENSSSCG00000000795
5	250054	250054	exonic	nonsynonymous SNV	ENSSSCG00000000800
5	710303	710303	exonic	nonsynonymous SNV	ENSSSCG00000000804
5	710641	710641	exonic	nonsynonymous SNV	ENSSSCG00000000810
5	3200392	3200392	exonic	nonsynonymous SNV	ENSSSCG00000000811
5	3234791	3234791	exonic	nonsynonymous SNV	ENSSSCG00000043909
5	3969950	3969950	exonic	nonsynonymous SNV	ENSSSCG00000025523
5	4084833	4084833	exonic	nonsynonymous SNV	ENSSSCG00000035713
5	4650012	4650012	exonic	nonsynonymous SNV	ENSSSCG00000048328
5	5723569	5723569	exonic	nonsynonymous SNV	ENSSSCG00000000856
5	10771101	10771101	exonic	nonsynonymous SNV	ENSSSCG00000000859
5	13941777	13941777	exonic	nonsynonymous SNV	ENSSSCG00000000869

5	14173678	14173678	exonic	nonsynonymous SNV	ENSSSCG00000000874
5	15285726	15285726	exonic	nonsynonymous SNV	ENSSSCG00000000900
5	16076394	16076394	exonic	nonsynonymous SNV	ENSSSCG00000000918
5	16247802	16247802	exonic	nonsynonymous SNV	ENSSSCG00000000926
5	16310028	16310028	exonic	nonsynonymous SNV	ENSSSCG00000032321
5	17274064	17274064	exonic	nonsynonymous SNV	ENSSSCG00000000940
5	17358124	17358124	exonic	nonsynonymous SNV	ENSSSCG00000039541
5	17717949	17717949	exonic	nonsynonymous SNV	ENSSSCG00000035230
5	17731719	17731719	exonic	nonsynonymous SNV	ENSSSCG00000002653
5	18076501	18076501	exonic	nonsynonymous SNV	ENSSSCG00000034980
5	18115368	18115368	exonic	nonsynonymous SNV	ENSSSCG00000060235
5	18382065	18382065	exonic	nonsynonymous SNV	ENSSSCG00000039218
5	18488661	18488661	exonic	nonsynonymous SNV	ENSSSCG00000055534
5	18496467	18496467	exonic	nonsynonymous SNV	ENSSSCG00000002706
5	18753238	18753238	exonic	nonsynonymous SNV	ENSSSCG00000028878
5	20231579	20231579	exonic	nonsynonymous SNV	ENSSSCG00000002724
5	21635683	21635683	exonic	nonsynonymous SNV	ENSSSCG00000002754
5	22313044	22313044	exonic	nonsynonymous SNV	ENSSSCG00000025417
5	23086675	23086675	exonic	nonsynonymous SNV	ENSSSCG00000039751
5	23097309	23097309	exonic	nonsynonymous SNV	ENSSSCG00000021644
5	34792848	34792848	exonic	nonsynonymous SNV	ENSSSCG00000002862
5	36274520	36274520	exonic	nonsynonymous SNV	ENSSSCG00000002866
5	53765751	53765751	exonic	nonsynonymous SNV	ENSSSCG00000039166
5	53782941	53782941	exonic	nonsynonymous SNV	ENSSSCG00000002879
5	53784689	53784689	exonic	nonsynonymous SNV	ENSSSCG00000025126
5	57987013	57987013	exonic	nonsynonymous SNV	ENSSSCG00000021374
5	58092102	58092102	exonic	nonsynonymous SNV	ENSSSCG00000002888
5	59424375	59424375	exonic	nonsynonymous SNV	ENSSSCG00000025590
5	61228153	61228153	exonic	nonsynonymous SNV	ENSSSCG00000058790
5	61402744	61402744	exonic	nonsynonymous SNV	ENSSSCG00000002965
5	61652826	61652826	exonic	nonsynonymous SNV	ENSSSCG00000024281
5	61667790	61667790	exonic	nonsynonymous SNV	ENSSSCG00000021333
5	62368918	62368918	exonic	nonsynonymous SNV	ENSSSCG00000030048
5	62814184	62814184	exonic	nonsynonymous SNV	ENSSSCG00000027515
5	63543681	63543681	exonic	nonsynonymous SNV	ENSSSCG00000048377
5	63638709	63638709	exonic	nonsynonymous SNV	ENSSSCG00000021917
5	63970951	63970951	exonic	nonsynonymous SNV	ENSSSCG00000035487
5	66469783	66469783	exonic	nonsynonymous SNV	ENSSSCG00000033836
5	67633079	67633079	exonic	nonsynonymous SNV	ENSSSCG00000055235
5	67853223	67853223	exonic	nonsynonymous SNV	ENSSSCG00000032803
5	67855134	67855134	exonic	nonsynonymous SNV	ENSSSCG00000003040
5	68906246	68906246	exonic	nonsynonymous SNV	ENSSSCG00000003044
5	69065033	69065033	exonic	nonsynonymous SNV	ENSSSCG00000003048
5	69375114	69375114	exonic	nonsynonymous SNV	ENSSSCG00000003064

5	70962318	70962318	exonic	nonsynonymous SNV	ENSSSCG00000030637
5	71180061	71180061	exonic	nonsynonymous SNV	ENSSSCG00000003059
5	72871704	72871704	exonic	nonsynonymous SNV	ENSSSCG00000059115
5	74577842	74577842	exonic	nonsynonymous SNV	ENSSSCG00000032434
5	74818116	74818116	exonic	nonsynonymous SNV	ENSSSCG00000049211
5	76307142	76307142	exonic	nonsynonymous SNV	ENSSSCG00000003068
5	77643295	77643295	exonic	nonsynonymous SNV	ENSSSCG00000003073
5	77794654	77794654	exonic	nonsynonymous SNV	ENSSSCG00000003081
5	77794654	77794654	exonic	nonsynonymous SNV	ENSSSCG00000003092
5	78352409	78352409	exonic	nonsynonymous SNV	ENSSSCG00000003104
5	78985567	78985567	exonic	nonsynonymous SNV	ENSSSCG00000003110
5	79026200	79026200	exonic	nonsynonymous SNV	ENSSSCG00000024240
5	81391464	81391464	exonic	nonsynonymous SNV	ENSSSCG00000022774
5	82042419	82042419	exonic	nonsynonymous SNV	ENSSSCG00000026689
5	82828145	82828145	exonic	nonsynonymous SNV	ENSSSCG00000030561
5	83563499	83563499	exonic	nonsynonymous SNV	ENSSSCG00000003146
5	88067614	88067614	exonic	nonsynonymous SNV	ENSSSCG00000003198
5	91876831	91876831	exonic	nonsynonymous SNV	ENSSSCG00000003195
5	94385248	94385248	exonic	nonsynonymous SNV	ENSSSCG00000037710
5	96190582	96190582	exonic	nonsynonymous SNV	ENSSSCG00000024736
5	100138815	100138815	exonic	nonsynonymous SNV	ENSSSCG00000037466
6	603189	603189	exonic	nonsynonymous SNV	ENSSSCG00000003232
6	959857	959857	exonic	nonsynonymous SNV	ENSSSCG00000022675
6	966055	966055	exonic	nonsynonymous SNV	ENSSSCG00000038287
6	1730795	1730795	exonic	nonsynonymous SNV	ENSSSCG00000024567
6	3042453	3042453	exonic	nonsynonymous SNV	ENSSSCG00000003302
6	4415328	4415328	exonic	nonsynonymous SNV	ENSSSCG00000003301
6	4587552	4587552	exonic	nonsynonymous SNV	ENSSSCG00000051187
6	12020903	12020903	exonic	nonsynonymous SNV	ENSSSCG00000025343
6	12099889	12099889	exonic	nonsynonymous SNV	ENSSSCG00000037450
6	12404001	12404001	exonic	nonsynonymous SNV	ENSSSCG00000044019
6	13215495	13215495	exonic	nonsynonymous SNV	ENSSSCG00000042915
6	17296094	17296094	exonic	nonsynonymous SNV	ENSSSCG00000003987
6	18551089	18551089	exonic	nonsynonymous SNV	ENSSSCG00000003348
6	18982314	18982314	exonic	nonsynonymous SNV	ENSSSCG00000034547
6	34519443	34519443	exonic	nonsynonymous SNV	ENSSSCG00000024678
6	42942517	42942517	exonic	nonsynonymous SNV	ENSSSCG00000003360
6	43085128	43085128	exonic	nonsynonymous SNV	ENSSSCG00000003362
6	44151308	44151308	exonic	nonsynonymous SNV	ENSSSCG00000003382
6	44591691	44591691	exonic	nonsynonymous SNV	ENSSSCG00000003410
6	44657643	44657643	exonic	nonsynonymous SNV	ENSSSCG00000003468
6	44672745	44672745	exonic	nonsynonymous SNV	ENSSSCG00000003466
6	44973250	44973250	exonic	nonsynonymous SNV	ENSSSCG00000003478
6	46543285	46543285	exonic	nonsynonymous SNV	ENSSSCG00000039973

6	47372942	47372942	exonic	nonsynonymous SNV	ENSSSCG00000003508
6	47442721	47442721	exonic	nonsynonymous SNV	ENSSSCG00000003513
6	47554015	47554015	exonic	nonsynonymous SNV	ENSSSCG00000003514
6	47590711	47590711	exonic	nonsynonymous SNV	ENSSSCG00000003521
6	47907080	47907080	exonic	nonsynonymous SNV	ENSSSCG00000003523
6	48077965	48077965	exonic	nonsynonymous SNV	ENSSSCG00000038706
6	48350569	48350569	exonic	nonsynonymous SNV	ENSSSCG00000028108
6	48477369	48477369	exonic	nonsynonymous SNV	ENSSSCG00000026025
6	48667091	48667091	exonic	nonsynonymous SNV	ENSSSCG00000036534
6	48668264	48668264	exonic	nonsynonymous SNV	ENSSSCG00000026526
6	48744599	48744599	exonic	nonsynonymous SNV	ENSSSCG00000057268
6	48816324	48816324	exonic	nonsynonymous SNV	ENSSSCG00000033539
6	48822143	48822143	exonic	nonsynonymous SNV	ENSSSCG00000003561
6	48929120	48929120	exonic	nonsynonymous SNV	ENSSSCG00000003586
6	49230766	49230766	exonic	nonsynonymous SNV	ENSSSCG00000058017
6	49987062	49987062	exonic	nonsynonymous SNV	ENSSSCG00000003603
6	50021899	50021899	exonic	nonsynonymous SNV	ENSSSCG00000022014
6	50055915	50055915	exonic	nonsynonymous SNV	ENSSSCG00000040173
6	50376209	50376209	exonic	nonsynonymous SNV	ENSSSCG00000037699
6	50393435	50393435	exonic	nonsynonymous SNV	ENSSSCG00000003644
6	50438078	50438078	exonic	nonsynonymous SNV	ENSSSCG00000003664
6	50438690	50438690	exonic	nonsynonymous SNV	ENSSSCG00000034765
6	50451358	50451358	exonic	nonsynonymous SNV	ENSSSCG00000039627
6	50451456	50451456	exonic	nonsynonymous SNV	ENSSSCG00000003687
6	50456166	50456166	exonic	nonsynonymous SNV	ENSSSCG00000003707
6	50503338	50503338	exonic	nonsynonymous SNV	ENSSSCG00000035965
6	50548061	50548061	exonic	nonsynonymous SNV	ENSSSCG00000003732
6	50549246	50549246	exonic	nonsynonymous SNV	ENSSSCG00000030018
6	50565752	50565752	exonic	nonsynonymous SNV	ENSSSCG00000038632
6	50608125	50608125	exonic	nonsynonymous SNV	ENSSSCG00000027421
6	50612307	50612307	exonic	nonsynonymous SNV	ENSSSCG00000027660
6	51224229	51224229	exonic	nonsynonymous SNV	ENSSSCG00000003766
6	52187302	52187302	exonic	nonsynonymous SNV	ENSSSCG00000003768
6	52604949	52604949	exonic	nonsynonymous SNV	ENSSSCG00000040109
6	52918539	52918539	exonic	nonsynonymous SNV	ENSSSCG00000003788
6	53076321	53076321	exonic	nonsynonymous SNV	ENSSSCG00000004829
6	53449815	53449815	exonic	nonsynonymous SNV	ENSSSCG00000003815
6	53742004	53742004	exonic	nonsynonymous SNV	ENSSSCG00000003821
6	53893178	53893178	exonic	nonsynonymous SNV	ENSSSCG00000003825
6	54013627	54013627	exonic	nonsynonymous SNV	ENSSSCG00000003831
6	54826143	54826143	exonic	nonsynonymous SNV	ENSSSCG00000003848
6	54875195	54875195	exonic	nonsynonymous SNV	ENSSSCG00000027119
6	55021089	55021089	exonic	nonsynonymous SNV	ENSSSCG00000003861
6	55541524	55541524	exonic	nonsynonymous SNV	ENSSSCG00000003868

6	55787330	55787330	exonic	nonsynonymous SNV	ENSSSCG00000046753
6	58488941	58488941	exonic	nonsynonymous SNV	ENSSSCG00000003875
6	59099105	59099105	exonic	nonsynonymous SNV	ENSSSCG00000003894
6	59280239	59280239	exonic	nonsynonymous SNV	ENSSSCG00000063244
6	59280240	59280240	exonic	nonsynonymous SNV	ENSSSCG00000038280
6	59393625	59393625	exonic	nonsynonymous SNV	ENSSSCG00000026793
6	59402363	59402363	exonic	nonsynonymous SNV	ENSSSCG00000039267
6	59414862	59414862	exonic	nonsynonymous SNV	ENSSSCG00000003941
6	59425540	59425540	exonic	nonsynonymous SNV	ENSSSCG00000023920
6	59436041	59436041	exonic	nonsynonymous SNV	ENSSSCG00000027374
6	59535840	59535840	exonic	nonsynonymous SNV	ENSSSCG00000003964
6	59636488	59636488	exonic	nonsynonymous SNV	ENSSSCG00000023266
6	59642984	59642984	exonic	nonsynonymous SNV	ENSSSCG00000037856
6	60389619	60389619	exonic	nonsynonymous SNV	ENSSSCG00000001008
6	60505286	60505286	exonic	nonsynonymous SNV	ENSSSCG00000036257
6	62329690	62329690	exonic	nonsynonymous SNV	ENSSSCG00000046603
6	63754923	63754923	exonic	nonsynonymous SNV	ENSSSCG00000026034
6	64480720	64480720	exonic	nonsynonymous SNV	ENSSSCG00000001087
6	65062481	65062481	exonic	nonsynonymous SNV	ENSSSCG00000001089
6	65155839	65155839	exonic	nonsynonymous SNV	ENSSSCG00000052503
6	65284501	65284501	exonic	nonsynonymous SNV	ENSSSCG00000001204
6	67404267	67404267	exonic	nonsynonymous SNV	ENSSSCG00000001207
6	71225720	71225720	exonic	nonsynonymous SNV	ENSSSCG00000022261
6	74902554	74902554	exonic	nonsynonymous SNV	ENSSSCG00000001232
6	74919715	74919715	exonic	nonsynonymous SNV	ENSSSCG00000024161
6	75579859	75579859	exonic	nonsynonymous SNV	ENSSSCG00000001342
6	77350920	77350920	exonic	nonsynonymous SNV	ENSSSCG00000031492
6	78903040	78903040	exonic	nonsynonymous SNV	ENSSSCG00000001362
6	78918159	78918159	exonic	nonsynonymous SNV	ENSSSCG00000001396
6	79450490	79450490	exonic	nonsynonymous SNV	ENSSSCG00000001409
6	79850827	79850827	exonic	nonsynonymous SNV	ENSSSCG00000034664
6	79866918	79866918	exonic	nonsynonymous SNV	ENSSSCG00000030368
6	79868997	79868997	exonic	nonsynonymous SNV	ENSSSCG00000061569
6	79871122	79871122	exonic	nonsynonymous SNV	ENSSSCG00000001455
6	79874642	79874642	exonic	nonsynonymous SNV	ENSSSCG00000001459
6	80112670	80112670	exonic	nonsynonymous SNV	ENSSSCG00000001484
6	80545607	80545607	exonic	nonsynonymous SNV	ENSSSCG00000001504
6	80596764	80596764	exonic	nonsynonymous SNV	ENSSSCG00000001506
6	81319455	81319455	exonic	nonsynonymous SNV	ENSSSCG00000001518
6	81613526	81613526	exonic	nonsynonymous SNV	ENSSSCG00000001534
6	81849926	81849926	exonic	nonsynonymous SNV	ENSSSCG00000001549
6	83609522	83609522	exonic	nonsynonymous SNV	ENSSSCG00000056995
6	83725330	83725330	exonic	nonsynonymous SNV	ENSSSCG00000001592
6	83740164	83740164	exonic	nonsynonymous SNV	ENSSSCG00000001594

6	83757411	83757411	exonic	nonsynonymous SNV	ENSSSCG00000036801
6	84177737	84177737	exonic	nonsynonymous SNV	ENSSSCG00000001651
6	85945329	85945329	exonic	nonsynonymous SNV	ENSSSCG00000001664
6	86030473	86030473	exonic	nonsynonymous SNV	ENSSSCG00000001720
6	88220096	88220096	exonic	nonsynonymous SNV	ENSSSCG00000001724
6	88689478	88689478	exonic	nonsynonymous SNV	ENSSSCG00000025535
6	91142142	91142142	exonic	nonsynonymous SNV	ENSSSCG00000002620
6	91833916	91833916	exonic	nonsynonymous SNV	ENSSSCG00000032430
6	93982708	93982708	exonic	nonsynonymous SNV	ENSSSCG00000038491
6	95554349	95554349	exonic	nonsynonymous SNV	ENSSSCG00000041322
6	97905117	97905117	exonic	nonsynonymous SNV	ENSSSCG00000036356
6	101215311	101215311	exonic	nonsynonymous SNV	ENSSSCG00000001817
6	101577152	101577152	exonic	nonsynonymous SNV	ENSSSCG00000001822
6	101667967	101667967	exonic	nonsynonymous SNV	ENSSSCG00000001823
6	108411220	108411220	exonic	nonsynonymous SNV	ENSSSCG00000030626
6	111418637	111418637	exonic	nonsynonymous SNV	ENSSSCG00000034559
6	116035585	116035585	exonic	nonsynonymous SNV	ENSSSCG00000032460
6	119190098	119190098	exonic	nonsynonymous SNV	ENSSSCG00000001873
6	128109811	128109811	exonic	nonsynonymous SNV	ENSSSCG00000001920
6	133596973	133596973	exonic	nonsynonymous SNV	ENSSSCG00000001930
6	134670533	134670533	exonic	nonsynonymous SNV	ENSSSCG00000001988
6	135245220	135245220	exonic	nonsynonymous SNV	ENSSSCG00000002037
6	135344058	135344058	exonic	nonsynonymous SNV	ENSSSCG00000022057
6	138828959	138828959	exonic	nonsynonymous SNV	ENSSSCG00000023790
6	141959934	141959934	exonic	nonsynonymous SNV	ENSSSCG00000059608
6	147838538	147838538	exonic	nonsynonymous SNV	ENSSSCG00000060054
6	149065786	149065786	exonic	nonsynonymous SNV	ENSSSCG00000052168
6	150157665	150157665	exonic	nonsynonymous SNV	ENSSSCG00000036662
6	150158065	150158065	exonic	nonsynonymous SNV	ENSSSCG00000002298
6	152432391	152432391	exonic	nonsynonymous SNV	ENSSSCG00000002332
6	153949277	153949277	exonic	nonsynonymous SNV	ENSSSCG00000056471
6	158970351	158970351	exonic	nonsynonymous SNV	ENSSSCG00000002368
6	158978413	158978413	exonic	nonsynonymous SNV	ENSSSCG00000002386
6	159515757	159515757	exonic	nonsynonymous SNV	ENSSSCG00000002391
6	159838620	159838620	exonic	nonsynonymous SNV	ENSSSCG00000024377
6	160156050	160156050	exonic	nonsynonymous SNV	ENSSSCG00000002446
6	160156050	160156050	exonic	nonsynonymous SNV	ENSSSCG00000002457
6	160156066	160156066	exonic	nonsynonymous SNV	ENSSSCG00000038515
6	160156066	160156066	exonic	nonsynonymous SNV	ENSSSCG00000002478
6	160853449	160853449	exonic	nonsynonymous SNV	ENSSSCG00000030371
6	164719228	164719228	exonic	nonsynonymous SNV	ENSSSCG00000042191
6	164737478	164737478	exonic	nonsynonymous SNV	ENSSSCG00000027667
6	164952290	164952290	exonic	nonsynonymous SNV	ENSSSCG00000002512
6	165103218	165103218	exonic	nonsynonymous SNV	ENSSSCG00000027502

6	167313723	167313723	exonic	nonsynonymous SNV	ENSSSCG00000032808
6	167319814	167319814	exonic	nonsynonymous SNV	ENSSSCG00000037035
6	167830508	167830508	exonic	nonsynonymous SNV	ENSSSCG00000008698
6	167833835	167833835	exonic	nonsynonymous SNV	ENSSSCG00000008722
6	168668375	168668375	exonic	nonsynonymous SNV	ENSSSCG00000034286
6	168668375	168668375	exonic	nonsynonymous SNV	ENSSSCG00000008709
6	168750364	168750364	exonic	nonsynonymous SNV	ENSSSCG00000024399
6	169047928	169047928	exonic	nonsynonymous SNV	ENSSSCG00000008735
6	169752485	169752485	exonic	nonsynonymous SNV	ENSSSCG00000038410
7	1909580	1909580	exonic	nonsynonymous SNV	ENSSSCG00000008738
7	5386031	5386031	exonic	nonsynonymous SNV	ENSSSCG00000048996
7	10592677	10592677	exonic	nonsynonymous SNV	ENSSSCG00000028632
7	13445168	13445168	exonic	nonsynonymous SNV	ENSSSCG00000008768
7	19244903	19244903	exonic	nonsynonymous SNV	ENSSSCG00000028471
7	19328916	19328916	exonic	nonsynonymous SNV	ENSSSCG00000008769
7	21885631	21885631	exonic	nonsynonymous SNV	ENSSSCG00000028983
7	22101124	22101124	exonic	nonsynonymous SNV	ENSSSCG00000029385
7	22155416	22155416	exonic	nonsynonymous SNV	ENSSSCG00000045169
7	22668717	22668717	exonic	nonsynonymous SNV	ENSSSCG00000008799
7	22799046	22799046	exonic	nonsynonymous SNV	ENSSSCG00000008812
7	22975496	22975496	exonic	nonsynonymous SNV	ENSSSCG00000008816
7	23058016	23058016	exonic	nonsynonymous SNV	ENSSSCG00000037950
7	23219093	23219093	exonic	nonsynonymous SNV	ENSSSCG00000008820
7	23236795	23236795	exonic	nonsynonymous SNV	ENSSSCG00000053955
7	23622846	23622846	exonic	nonsynonymous SNV	ENSSSCG00000036417
7	23752057	23752057	exonic	nonsynonymous SNV	ENSSSCG00000051162
7	23752565	23752565	exonic	nonsynonymous SNV	ENSSSCG00000008870
7	23835601	23835601	exonic	nonsynonymous SNV	ENSSSCG00000008873
7	23912062	23912062	exonic	nonsynonymous SNV	ENSSSCG00000039175
7	24882411	24882411	exonic	nonsynonymous SNV	ENSSSCG00000038890
7	24901590	24901590	exonic	nonsynonymous SNV	ENSSSCG00000022283
7	25032843	25032843	exonic	nonsynonymous SNV	ENSSSCG00000047476
7	26485949	26485949	exonic	nonsynonymous SNV	ENSSSCG00000053199
7	29646260	29646260	exonic	nonsynonymous SNV	ENSSSCG00000025514
7	29656246	29656246	exonic	nonsynonymous SNV	ENSSSCG00000027609
7	29889211	29889211	exonic	nonsynonymous SNV	ENSSSCG00000008948
7	30898887	30898887	exonic	nonsynonymous SNV	ENSSSCG00000008973
7	31543474	31543474	exonic	nonsynonymous SNV	ENSSSCG00000008993
7	34555423	34555423	exonic	nonsynonymous SNV	ENSSSCG00000026886
7	34717614	34717614	exonic	nonsynonymous SNV	ENSSSCG00000009001
7	34762031	34762031	exonic	nonsynonymous SNV	ENSSSCG00000009011
7	37225946	37225946	exonic	nonsynonymous SNV	ENSSSCG00000020879
7	38022046	38022046	exonic	nonsynonymous SNV	ENSSSCG00000058219
7	38314468	38314468	exonic	nonsynonymous SNV	ENSSSCG00000026360

7	41436326	41436326	exonic	nonsynonymous SNV	ENSSSCG00000030396
7	41619630	41619630	exonic	nonsynonymous SNV	ENSSSCG00000029943
7	44750460	44750460	exonic	nonsynonymous SNV	ENSSSCG00000022173
7	46318596	46318596	exonic	nonsynonymous SNV	ENSSSCG00000009090
7	48416894	48416894	exonic	nonsynonymous SNV	ENSSSCG00000009096
7	50750550	50750550	exonic	nonsynonymous SNV	ENSSSCG00000029260
7	52676025	52676025	exonic	nonsynonymous SNV	ENSSSCG00000009114
7	52955193	52955193	exonic	nonsynonymous SNV	ENSSSCG00000009136
7	53464633	53464633	exonic	nonsynonymous SNV	ENSSSCG00000009140
7	53471128	53471128	exonic	nonsynonymous SNV	ENSSSCG00000039813
7	53549042	53549042	exonic	nonsynonymous SNV	ENSSSCG00000022788
7	53592784	53592784	exonic	nonsynonymous SNV	ENSSSCG00000023296
7	53750767	53750767	exonic	nonsynonymous SNV	ENSSSCG00000009176
7	54125117	54125117	exonic	nonsynonymous SNV	ENSSSCG00000062441
7	54200859	54200859	exonic	nonsynonymous SNV	ENSSSCG00000051020
7	57972261	57972261	exonic	nonsynonymous SNV	ENSSSCG00000009184
7	60010834	60010834	exonic	nonsynonymous SNV	ENSSSCG00000022353
7	61026083	61026083	exonic	nonsynonymous SNV	ENSSSCG00000022986
7	74971451	74971451	exonic	nonsynonymous SNV	ENSSSCG00000038867
7	75999954	75999954	exonic	nonsynonymous SNV	ENSSSCG00000026655
7	76857608	76857608	exonic	nonsynonymous SNV	ENSSSCG00000009230
7	77038682	77038682	exonic	nonsynonymous SNV	ENSSSCG00000014581
7	78250104	78250104	exonic	nonsynonymous SNV	ENSSSCG00000014587
7	79587214	79587214	exonic	nonsynonymous SNV	ENSSSCG00000059225
7	79996720	79996720	exonic	nonsynonymous SNV	ENSSSCG00000056036
7	80427403	80427403	exonic	nonsynonymous SNV	ENSSSCG00000036687
7	91541283	91541283	exonic	nonsynonymous SNV	ENSSSCG00000057177
7	95024161	95024161	exonic	nonsynonymous SNV	ENSSSCG00000060093
7	96900825	96900825	exonic	nonsynonymous SNV	ENSSSCG00000061789
7	97784613	97784613	exonic	nonsynonymous SNV	ENSSSCG00000063516
7	99223597	99223597	exonic	nonsynonymous SNV	ENSSSCG00000049272
7	99993320	99993320	exonic	nonsynonymous SNV	ENSSSCG00000044470
7	112753039	112753039	exonic	nonsynonymous SNV	ENSSSCG00000021619
7	113636715	113636715	exonic	nonsynonymous SNV	ENSSSCG00000014598
7	113642456	113642456	exonic	nonsynonymous SNV	ENSSSCG00000029189
7	114367935	114367935	exonic	nonsynonymous SNV	ENSSSCG00000055267
7	115609284	115609284	exonic	nonsynonymous SNV	ENSSSCG00000021919
7	115729379	115729379	exonic	nonsynonymous SNV	ENSSSCG00000031506
7	115851949	115851949	exonic	nonsynonymous SNV	ENSSSCG00000055108
7	120260741	120260741	exonic	nonsynonymous SNV	ENSSSCG00000060652
7	120834511	120834511	exonic	nonsynonymous SNV	ENSSSCG00000014728
7	121061152	121061152	exonic	nonsynonymous SNV	ENSSSCG00000014759
8	120271	120271	exonic	nonsynonymous SNV	ENSSSCG00000039656
8	416300	416300	exonic	nonsynonymous SNV	ENSSSCG00000014835

8	590153	590153	exonic	nonsynonymous SNV	ENSSSCG00000014897
8	1989650	1989650	exonic	nonsynonymous SNV	ENSSSCG00000014909
8	2868743	2868743	exonic	nonsynonymous SNV	ENSSSCG00000014920
8	4437621	4437621	exonic	nonsynonymous SNV	ENSSSCG00000046061
8	4643825	4643825	exonic	nonsynonymous SNV	ENSSSCG00000014934
8	4644747	4644747	exonic	nonsynonymous SNV	ENSSSCG00000014952
8	4678451	4678451	exonic	nonsynonymous SNV	ENSSSCG00000014977
8	4982277	4982277	exonic	nonsynonymous SNV	ENSSSCG00000015001
8	5003201	5003201	exonic	nonsynonymous SNV	ENSSSCG00000015009
8	9228033	9228033	exonic	nonsynonymous SNV	ENSSSCG00000015010
8	10401565	10401565	exonic	nonsynonymous SNV	ENSSSCG00000015018
8	10874177	10874177	exonic	nonsynonymous SNV	ENSSSCG00000015039
8	10896504	10896504	exonic	nonsynonymous SNV	ENSSSCG00000015080
8	18052929	18052929	exonic	nonsynonymous SNV	ENSSSCG00000015082
8	19450420	19450420	exonic	nonsynonymous SNV	ENSSSCG00000023324
8	19450779	19450779	exonic	nonsynonymous SNV	ENSSSCG00000015099
8	27798215	27798215	exonic	nonsynonymous SNV	ENSSSCG00000020685
8	28024893	28024893	exonic	nonsynonymous SNV	ENSSSCG00000015202
8	28034289	28034289	exonic	nonsynonymous SNV	ENSSSCG00000015203
8	28306579	28306579	exonic	nonsynonymous SNV	ENSSSCG00000015227
8	29483571	29483571	exonic	nonsynonymous SNV	ENSSSCG00000015237
8	29548972	29548972	exonic	nonsynonymous SNV	ENSSSCG00000015246
8	30708680	30708680	exonic	nonsynonymous SNV	ENSSSCG00000015250
8	31594939	31594939	exonic	nonsynonymous SNV	ENSSSCG00000032446
8	32707567	32707567	exonic	nonsynonymous SNV	ENSSSCG00000015294
8	32716380	32716380	exonic	nonsynonymous SNV	ENSSSCG00000021276
8	37506211	37506211	exonic	nonsynonymous SNV	ENSSSCG00000015310
8	37875024	37875024	exonic	nonsynonymous SNV	ENSSSCG00000022982
8	37955577	37955577	exonic	nonsynonymous SNV	ENSSSCG00000015328
8	38053512	38053512	exonic	nonsynonymous SNV	ENSSSCG00000047863
8	42150769	42150769	exonic	nonsynonymous SNV	ENSSSCG00000038187
8	42514532	42514532	exonic	nonsynonymous SNV	ENSSSCG00000015342
8	44146944	44146944	exonic	nonsynonymous SNV	ENSSSCG00000015346
8	44587713	44587713	exonic	nonsynonymous SNV	ENSSSCG00000022543
8	47096586	47096586	exonic	nonsynonymous SNV	ENSSSCG00000049585
8	52471535	52471535	exonic	nonsynonymous SNV	ENSSSCG00000015353
8	53603355	53603355	exonic	nonsynonymous SNV	ENSSSCG00000015379
8	55255694	55255694	exonic	nonsynonymous SNV	ENSSSCG00000015386
8	66235680	66235680	exonic	nonsynonymous SNV	ENSSSCG00000032241
8	66474601	66474601	exonic	nonsynonymous SNV	ENSSSCG00000024520
8	67551553	67551553	exonic	nonsynonymous SNV	ENSSSCG00000015430
8	68335846	68335846	exonic	nonsynonymous SNV	ENSSSCG00000054021
8	69582988	69582988	exonic	nonsynonymous SNV	ENSSSCG00000015432
8	71574630	71574630	exonic	nonsynonymous SNV	ENSSSCG00000015445

8	74041390	74041390	exonic	nonsynonymous SNV	ENSSSCG00000015450
8	74933544	74933544	exonic	nonsynonymous SNV	ENSSSCG00000015477
8	75433065	75433065	exonic	nonsynonymous SNV	ENSSSCG00000015509
8	75433619	75433619	exonic	nonsynonymous SNV	ENSSSCG00000015535
8	76108922	76108922	exonic	nonsynonymous SNV	ENSSSCG00000015548
8	76116693	76116693	exonic	nonsynonymous SNV	ENSSSCG00000015555
8	76670439	76670439	exonic	nonsynonymous SNV	ENSSSCG00000035595
8	78168208	78168208	exonic	nonsynonymous SNV	ENSSSCG00000015602
8	86666759	86666759	exonic	nonsynonymous SNV	ENSSSCG00000038066
8	87478888	87478888	exonic	nonsynonymous SNV	ENSSSCG00000010829
8	87512708	87512708	exonic	nonsynonymous SNV	ENSSSCG00000034164
8	95977815	95977815	exonic	nonsynonymous SNV	ENSSSCG00000031212
8	99821926	99821926	exonic	nonsynonymous SNV	ENSSSCG00000010879
8	101767586	101767586	exonic	nonsynonymous SNV	ENSSSCG00000010885
8	102272042	102272042	exonic	nonsynonymous SNV	ENSSSCG00000021897
8	103086106	103086106	exonic	nonsynonymous SNV	ENSSSCG00000024784
8	105122093	105122093	exonic	nonsynonymous SNV	ENSSSCG00000039843
8	112384602	112384602	exonic	nonsynonymous SNV	ENSSSCG00000034151
8	112533138	112533138	exonic	nonsynonymous SNV	ENSSSCG00000010926
8	113101595	113101595	exonic	nonsynonymous SNV	ENSSSCG00000040707
8	116277375	116277375	exonic	nonsynonymous SNV	ENSSSCG00000039342
8	117925371	117925371	exonic	nonsynonymous SNV	ENSSSCG00000033196
8	117925410	117925410	exonic	nonsynonymous SNV	ENSSSCG00000024384
8	117931134	117931134	exonic	nonsynonymous SNV	ENSSSCG00000010990
8	120638982	120638982	exonic	nonsynonymous SNV	ENSSSCG00000035454
8	121027789	121027789	exonic	nonsynonymous SNV	ENSSSCG00000030408
8	121027960	121027960	exonic	nonsynonymous SNV	ENSSSCG00000011070
8	121198716	121198716	exonic	nonsynonymous SNV	ENSSSCG00000055608
8	121229998	121229998	exonic	nonsynonymous SNV	ENSSSCG00000022045
8	121975385	121975385	exonic	nonsynonymous SNV	ENSSSCG00000038007
8	128525336	128525336	exonic	nonsynonymous SNV	ENSSSCG00000047783
8	130759230	130759230	exonic	nonsynonymous SNV	ENSSSCG00000011046
8	132077484	132077484	exonic	nonsynonymous SNV	ENSSSCG00000039658
8	132081385	132081385	exonic	nonsynonymous SNV	ENSSSCG00000039244
8	133891633	133891633	exonic	nonsynonymous SNV	ENSSSCG00000011068
9	1192852	1192852	exonic	nonsynonymous SNV	ENSSSCG00000011060
9	1410737	1410737	exonic	nonsynonymous SNV	ENSSSCG00000038536
9	1455408	1455408	exonic	nonsynonymous SNV	ENSSSCG00000011074
9	1474728	1474728	exonic	nonsynonymous SNV	ENSSSCG00000011078
9	1540201	1540201	exonic	nonsynonymous SNV	ENSSSCG00000011080
9	1540503	1540503	exonic	nonsynonymous SNV	ENSSSCG00000040615
9	1662294	1662294	exonic	nonsynonymous SNV	ENSSSCG00000011102
9	1678076	1678076	exonic	nonsynonymous SNV	ENSSSCG00000011103
9	1847190	1847190	exonic	nonsynonymous SNV	ENSSSCG00000011114

9	1869207	1869207	exonic	nonsynonymous SNV	ENSSSCG00000039539
9	1900313	1900313	exonic	nonsynonymous SNV	ENSSSCG00000023105
9	1917107	1917107	exonic	nonsynonymous SNV	ENSSSCG00000011162
9	1948335	1948335	exonic	nonsynonymous SNV	ENSSSCG00000035493
9	2023335	2023335	exonic	nonsynonymous SNV	ENSSSCG00000023771
9	3113965	3113965	exonic	nonsynonymous SNV	ENSSSCG00000009308
9	3114397	3114397	exonic	nonsynonymous SNV	ENSSSCG00000009313
9	3489589	3489589	exonic	nonsynonymous SNV	ENSSSCG00000025996
9	4131723	4131723	exonic	nonsynonymous SNV	ENSSSCG00000028065
9	4192951	4192951	exonic	nonsynonymous SNV	ENSSSCG00000009338
9	4706132	4706132	exonic	nonsynonymous SNV	ENSSSCG00000046579
9	4737371	4737371	exonic	nonsynonymous SNV	ENSSSCG00000051344
9	4845593	4845593	exonic	nonsynonymous SNV	ENSSSCG00000009357
9	5330541	5330541	exonic	nonsynonymous SNV	ENSSSCG00000056668
9	6283243	6283243	exonic	nonsynonymous SNV	ENSSSCG00000009364
9	8464824	8464824	exonic	nonsynonymous SNV	ENSSSCG00000009411
9	16922608	16922608	exonic	nonsynonymous SNV	ENSSSCG00000009413
9	16922968	16922968	exonic	nonsynonymous SNV	ENSSSCG00000031627
9	19647101	19647101	exonic	nonsynonymous SNV	ENSSSCG00000025120
9	20718786	20718786	exonic	nonsynonymous SNV	ENSSSCG00000049910
9	22998767	22998767	exonic	nonsynonymous SNV	ENSSSCG00000050522
9	23194069	23194069	exonic	nonsynonymous SNV	ENSSSCG00000035248
9	26520311	26520311	exonic	nonsynonymous SNV	ENSSSCG00000009486
9	26520610	26520610	exonic	nonsynonymous SNV	ENSSSCG00000044700
9	32127968	32127968	exonic	nonsynonymous SNV	ENSSSCG00000009545
9	36296422	36296422	exonic	nonsynonymous SNV	ENSSSCG00000031757
9	36862115	36862115	exonic	nonsynonymous SNV	ENSSSCG00000055162
9	36866146	36866146	exonic	nonsynonymous SNV	ENSSSCG00000017146
9	36893382	36893382	exonic	nonsynonymous SNV	ENSSSCG00000031600
9	39059825	39059825	exonic	nonsynonymous SNV	ENSSSCG00000021342
9	39878369	39878369	exonic	nonsynonymous SNV	ENSSSCG00000017200
9	44729740	44729740	exonic	nonsynonymous SNV	ENSSSCG00000055960
9	44882016	44882016	exonic	nonsynonymous SNV	ENSSSCG00000062259
9	45804272	45804272	exonic	nonsynonymous SNV	ENSSSCG00000023612
9	46151985	46151985	exonic	nonsynonymous SNV	ENSSSCG00000023014
9	46153802	46153802	exonic	nonsynonymous SNV	ENSSSCG00000024059
9	51127035	51127035	exonic	nonsynonymous SNV	ENSSSCG00000035199
9	52078598	52078598	exonic	nonsynonymous SNV	ENSSSCG00000017389
9	52084362	52084362	exonic	nonsynonymous SNV	ENSSSCG00000021910
9	53362338	53362338	exonic	nonsynonymous SNV	ENSSSCG00000033085
9	55743534	55743534	exonic	nonsynonymous SNV	ENSSSCG00000032478
9	56919560	56919560	exonic	nonsynonymous SNV	ENSSSCG00000017563
9	57149542	57149542	exonic	nonsynonymous SNV	ENSSSCG00000017693
9	67649483	67649483	exonic	nonsynonymous SNV	ENSSSCG00000026596

9	68113776	68113776	exonic	nonsynonymous SNV	ENSSSCG00000031292
9	69018337	69018337	exonic	nonsynonymous SNV	ENSSSCG00000031114
9	72050632	72050632	exonic	nonsynonymous SNV	ENSSSCG00000017717
9	73205605	73205605	exonic	nonsynonymous SNV	ENSSSCG00000040177
9	74433021	74433021	exonic	nonsynonymous SNV	ENSSSCG00000017753
9	76252061	76252061	exonic	nonsynonymous SNV	ENSSSCG00000017755
9	77532125	77532125	exonic	nonsynonymous SNV	ENSSSCG00000023478
9	77691779	77691779	exonic	nonsynonymous SNV	ENSSSCG00000017795
9	77767212	77767212	exonic	nonsynonymous SNV	ENSSSCG00000017799
9	78487756	78487756	exonic	nonsynonymous SNV	ENSSSCG00000028078
9	81956240	81956240	exonic	nonsynonymous SNV	ENSSSCG00000017832
9	82076533	82076533	exonic	nonsynonymous SNV	ENSSSCG00000034858
9	82184489	82184489	exonic	nonsynonymous SNV	ENSSSCG00000038664
9	90693731	90693731	exonic	nonsynonymous SNV	ENSSSCG00000054106
9	90741238	90741238	exonic	nonsynonymous SNV	ENSSSCG00000031932
9	91761901	91761901	exonic	nonsynonymous SNV	ENSSSCG00000038683
9	92049198	92049198	exonic	nonsynonymous SNV	ENSSSCG00000056589
9	93178676	93178676	exonic	nonsynonymous SNV	ENSSSCG00000017868
9	93260496	93260496	exonic	nonsynonymous SNV	ENSSSCG00000017869
9	105425107	105425107	exonic	nonsynonymous SNV	ENSSSCG00000017873
9	105425107	105425107	exonic	nonsynonymous SNV	ENSSSCG00000032049
9	105642779	105642779	exonic	nonsynonymous SNV	ENSSSCG00000033819
9	107971963	107971963	exonic	nonsynonymous SNV	ENSSSCG00000022026
9	109245504	109245504	exonic	nonsynonymous SNV	ENSSSCG00000017897
9	113937983	113937983	exonic	nonsynonymous SNV	ENSSSCG00000024584
9	117967142	117967142	exonic	nonsynonymous SNV	ENSSSCG00000040188
9	121805292	121805292	exonic	nonsynonymous SNV	ENSSSCG00000017962
9	123857108	123857108	exonic	nonsynonymous SNV	ENSSSCG00000025345
9	124397331	124397331	exonic	nonsynonymous SNV	ENSSSCG00000060339
9	127245411	127245411	exonic	nonsynonymous SNV	ENSSSCG00000017983
9	131322858	131322858	exonic	nonsynonymous SNV	ENSSSCG00000028788
10	6472313	6472313	exonic	nonsynonymous SNV	ENSSSCG00000017998
10	10191122	10191122	exonic	nonsynonymous SNV	ENSSSCG00000018005
10	11825456	11825456	exonic	nonsynonymous SNV	ENSSSCG00000029441
10	11839527	11839527	exonic	nonsynonymous SNV	ENSSSCG00000018023
10	18194206	18194206	exonic	nonsynonymous SNV	ENSSSCG00000018039
10	19391571	19391571	exonic	nonsynonymous SNV	ENSSSCG00000034364
10	20269177	20269177	exonic	nonsynonymous SNV	ENSSSCG00000024030
10	23515475	23515475	exonic	nonsynonymous SNV	ENSSSCG00000018046
10	24126467	24126467	exonic	nonsynonymous SNV	ENSSSCG00000039961
10	24234218	24234218	exonic	nonsynonymous SNV	ENSSSCG00000035839
10	24645694	24645694	exonic	nonsynonymous SNV	ENSSSCG00000038164
10	31994324	31994324	exonic	nonsynonymous SNV	ENSSSCG00000018055
10	32297131	32297131	exonic	nonsynonymous SNV	ENSSSCG00000051348

10	32440750	32440750	exonic	nonsynonymous SNV	ENSSSCG00000011179
10	32700318	32700318	exonic	nonsynonymous SNV	ENSSSCG00000011181
10	33025694	33025694	exonic	nonsynonymous SNV	ENSSSCG00000011213
10	33317682	33317682	exonic	nonsynonymous SNV	ENSSSCG00000011214
10	33902730	33902730	exonic	nonsynonymous SNV	ENSSSCG00000039358
10	33902813	33902813	exonic	nonsynonymous SNV	ENSSSCG00000025502
10	39535178	39535178	exonic	nonsynonymous SNV	ENSSSCG00000011239
10	42967281	42967281	exonic	nonsynonymous SNV	ENSSSCG00000011243
10	42987406	42987406	exonic	nonsynonymous SNV	ENSSSCG00000011245
10	43929299	43929299	exonic	nonsynonymous SNV	ENSSSCG00000025783
10	44543242	44543242	exonic	nonsynonymous SNV	ENSSSCG00000034200
10	46235275	46235275	exonic	nonsynonymous SNV	ENSSSCG00000039224
10	46949247	46949247	exonic	nonsynonymous SNV	ENSSSCG00000027992
10	48407692	48407692	exonic	nonsynonymous SNV	ENSSSCG00000011446
10	48719391	48719391	exonic	nonsynonymous SNV	ENSSSCG00000011461
10	48719767	48719767	exonic	nonsynonymous SNV	ENSSSCG00000011527
10	49388450	49388450	exonic	nonsynonymous SNV	ENSSSCG00000011531
10	49866523	49866523	exonic	nonsynonymous SNV	ENSSSCG00000023225
10	50663424	50663424	exonic	nonsynonymous SNV	ENSSSCG00000011645
10	51960103	51960103	exonic	nonsynonymous SNV	ENSSSCG00000011706
10	52509161	52509161	exonic	nonsynonymous SNV	ENSSSCG00000029240
10	55161166	55161166	exonic	nonsynonymous SNV	ENSSSCG00000011821
10	56288869	56288869	exonic	nonsynonymous SNV	ENSSSCG00000035414
10	57234624	57234624	exonic	nonsynonymous SNV	ENSSSCG00000011837
10	59774253	59774253	exonic	nonsynonymous SNV	ENSSSCG00000023527
10	59853528	59853528	exonic	nonsynonymous SNV	ENSSSCG00000011846
10	65459341	65459341	exonic	nonsynonymous SNV	ENSSSCG00000011867
10	68663717	68663717	exonic	nonsynonymous SNV	ENSSSCG00000037882
11	2408781	2408781	exonic	nonsynonymous SNV	ENSSSCG00000025436
11	3883210	3883210	exonic	nonsynonymous SNV	ENSSSCG00000058360
11	4977864	4977864	exonic	nonsynonymous SNV	ENSSSCG00000022081
11	5194965	5194965	exonic	nonsynonymous SNV	ENSSSCG00000011933
11	6199016	6199016	exonic	nonsynonymous SNV	ENSSSCG00000011942
11	6199056	6199056	exonic	nonsynonymous SNV	ENSSSCG00000030486
11	6199233	6199233	exonic	nonsynonymous SNV	ENSSSCG00000060380
11	7359866	7359866	exonic	nonsynonymous SNV	ENSSSCG00000029392
11	8613914	8613914	exonic	nonsynonymous SNV	ENSSSCG00000012054
11	9690509	9690509	exonic	nonsynonymous SNV	ENSSSCG00000012062
11	11448570	11448570	exonic	nonsynonymous SNV	ENSSSCG00000021042
11	12718757	12718757	exonic	nonsynonymous SNV	ENSSSCG00000012076
11	13770269	13770269	exonic	nonsynonymous SNV	ENSSSCG00000025821
11	13960040	13960040	exonic	nonsynonymous SNV	ENSSSCG00000029627
11	21091946	21091946	exonic	nonsynonymous SNV	ENSSSCG00000025133
11	21293629	21293629	exonic	nonsynonymous SNV	ENSSSCG00000033204

11	25735462	25735462	exonic	nonsynonymous SNV	ENSSSCG00000028559
11	45032994	45032994	exonic	nonsynonymous SNV	ENSSSCG00000009638
11	46211232	46211232	exonic	nonsynonymous SNV	ENSSSCG00000033037
11	52356374	52356374	exonic	nonsynonymous SNV	ENSSSCG00000009659
11	55327968	55327968	exonic	nonsynonymous SNV	ENSSSCG00000009664
11	56511546	56511546	exonic	nonsynonymous SNV	ENSSSCG00000009683
11	76942020	76942020	exonic	nonsynonymous SNV	ENSSSCG00000009685
11	77113800	77113800	exonic	nonsynonymous SNV	ENSSSCG00000009691
11	77264991	77264991	exonic	nonsynonymous SNV	ENSSSCG00000034740
12	2095660	2095660	exonic	nonsynonymous SNV	ENSSSCG00000026044
12	2121452	2121452	exonic	nonsynonymous SNV	ENSSSCG00000026659
12	2122932	2122932	exonic	nonsynonymous SNV	ENSSSCG00000032475
12	2138210	2138210	exonic	nonsynonymous SNV	ENSSSCG00000009705
12	2169770	2169770	exonic	nonsynonymous SNV	ENSSSCG00000042965
12	3797515	3797515	exonic	nonsynonymous SNV	ENSSSCG00000009720
12	5188158	5188158	exonic	nonsynonymous SNV	ENSSSCG00000035929
12	5586801	5586801	exonic	nonsynonymous SNV	ENSSSCG00000028018
12	11188060	11188060	exonic	nonsynonymous SNV	ENSSSCG00000009781
12	14986623	14986623	exonic	nonsynonymous SNV	ENSSSCG00000028889
12	15377777	15377777	exonic	nonsynonymous SNV	ENSSSCG00000009796
12	19282248	19282248	exonic	nonsynonymous SNV	ENSSSCG00000051618
12	19871937	19871937	exonic	nonsynonymous SNV	ENSSSCG00000009814
12	19909431	19909431	exonic	nonsynonymous SNV	ENSSSCG00000009873
12	20092602	20092602	exonic	nonsynonymous SNV	ENSSSCG00000009874
12	20953590	20953590	exonic	nonsynonymous SNV	ENSSSCG00000009876
12	21358760	21358760	exonic	nonsynonymous SNV	ENSSSCG00000009881
12	23384108	23384108	exonic	nonsynonymous SNV	ENSSSCG00000046640
12	26811471	26811471	exonic	nonsynonymous SNV	ENSSSCG00000038099
12	38462027	38462027	exonic	nonsynonymous SNV	ENSSSCG00000009896
12	39056439	39056439	exonic	nonsynonymous SNV	ENSSSCG00000009901
12	39388346	39388346	exonic	nonsynonymous SNV	ENSSSCG00000009912
12	39956800	39956800	exonic	nonsynonymous SNV	ENSSSCG00000009957
12	40021784	40021784	exonic	nonsynonymous SNV	ENSSSCG00000009968
12	40030795	40030795	exonic	nonsynonymous SNV	ENSSSCG00000035836
12	41727598	41727598	exonic	nonsynonymous SNV	ENSSSCG00000010077
12	44132776	44132776	exonic	nonsynonymous SNV	ENSSSCG00000010086
12	44190802	44190802	exonic	nonsynonymous SNV	ENSSSCG00000010089
12	44684331	44684331	exonic	nonsynonymous SNV	ENSSSCG00000010092
12	46212558	46212558	exonic	nonsynonymous SNV	ENSSSCG00000010093
12	46462337	46462337	exonic	nonsynonymous SNV	ENSSSCG00000010097
12	48153994	48153994	exonic	nonsynonymous SNV	ENSSSCG00000037762
12	48503465	48503465	exonic	nonsynonymous SNV	ENSSSCG00000010139
12	48503735	48503735	exonic	nonsynonymous SNV	ENSSSCG00000041446
12	48943486	48943486	exonic	nonsynonymous SNV	ENSSSCG00000010145

12	49103746	49103746	exonic	nonsynonymous SNV	ENSSSCG00000010146
12	49379481	49379481	exonic	nonsynonymous SNV	ENSSSCG00000010151
12	49389909	49389909	exonic	nonsynonymous SNV	ENSSSCG00000033692
12	49390256	49390256	exonic	nonsynonymous SNV	ENSSSCG00000034844
12	49390614	49390614	exonic	nonsynonymous SNV	ENSSSCG00000060488
12	49523676	49523676	exonic	nonsynonymous SNV	ENSSSCG00000010161
12	49544164	49544164	exonic	nonsynonymous SNV	ENSSSCG00000010175
12	49786710	49786710	exonic	nonsynonymous SNV	ENSSSCG00000023596
12	49830211	49830211	exonic	nonsynonymous SNV	ENSSSCG00000010212
12	49929164	49929164	exonic	nonsynonymous SNV	ENSSSCG00000028327
12	50108214	50108214	exonic	nonsynonymous SNV	ENSSSCG00000058274
12	50728045	50728045	exonic	nonsynonymous SNV	ENSSSCG00000010219
12	50915637	50915637	exonic	nonsynonymous SNV	ENSSSCG00000031360
12	51816884	51816884	exonic	nonsynonymous SNV	ENSSSCG00000010226
12	52729807	52729807	exonic	nonsynonymous SNV	ENSSSCG00000036480
12	52843265	52843265	exonic	nonsynonymous SNV	ENSSSCG00000029311
12	53105973	53105973	exonic	nonsynonymous SNV	ENSSSCG00000010239
12	53237427	53237427	exonic	nonsynonymous SNV	ENSSSCG00000010241
12	53335068	53335068	exonic	nonsynonymous SNV	ENSSSCG00000010266
12	53366056	53366056	exonic	nonsynonymous SNV	ENSSSCG00000036446
12	53366191	53366191	exonic	nonsynonymous SNV	ENSSSCG00000010273
12	53405835	53405835	exonic	nonsynonymous SNV	ENSSSCG00000010280
12	54725816	54725816	exonic	nonsynonymous SNV	ENSSSCG00000010278
12	55146905	55146905	exonic	nonsynonymous SNV	ENSSSCG00000010281
12	55267170	55267170	exonic	nonsynonymous SNV	ENSSSCG00000010293
12	58098834	58098834	exonic	nonsynonymous SNV	ENSSSCG00000050482
12	59333975	59333975	exonic	nonsynonymous SNV	ENSSSCG00000010316
12	59556313	59556313	exonic	nonsynonymous SNV	ENSSSCG00000048472
12	59758107	59758107	exonic	nonsynonymous SNV	ENSSSCG00000010331
12	59988473	59988473	exonic	nonsynonymous SNV	ENSSSCG00000010379
12	60161027	60161027	exonic	nonsynonymous SNV	ENSSSCG00000010401
12	60465514	60465514	exonic	nonsynonymous SNV	ENSSSCG00000010461
12	60762244	60762244	exonic	nonsynonymous SNV	ENSSSCG00000035635
12	60908868	60908868	exonic	nonsynonymous SNV	ENSSSCG00000010476
12	61501287	61501287	exonic	nonsynonymous SNV	ENSSSCG00000010480
13	985006	985006	exonic	nonsynonymous SNV	ENSSSCG00000061845
13	985156	985156	exonic	nonsynonymous SNV	ENSSSCG00000059716
13	1432339	1432339	exonic	nonsynonymous SNV	ENSSSCG00000010513
13	12511022	12511022	exonic	nonsynonymous SNV	ENSSSCG00000038904
13	12639988	12639988	exonic	nonsynonymous SNV	ENSSSCG00000010514
13	19043522	19043522	exonic	nonsynonymous SNV	ENSSSCG00000010528
13	21872625	21872625	exonic	nonsynonymous SNV	ENSSSCG00000010538
13	22016331	22016331	exonic	nonsynonymous SNV	ENSSSCG00000010541
13	22018884	22018884	exonic	nonsynonymous SNV	ENSSSCG00000006165

13	22206432	22206432	exonic	nonsynonymous SNV	ENSSSCG00000010575
13	22207938	22207938	exonic	nonsynonymous SNV	ENSSSCG00000010577
13	22387419	22387419	exonic	nonsynonymous SNV	ENSSSCG00000038856
13	24654154	24654154	exonic	nonsynonymous SNV	ENSSSCG00000010595
13	26099458	26099458	exonic	nonsynonymous SNV	ENSSSCG00000010607
13	30387599	30387599	exonic	nonsynonymous SNV	ENSSSCG00000032968
13	32417200	32417200	exonic	nonsynonymous SNV	ENSSSCG00000010651
13	34857100	34857100	exonic	nonsynonymous SNV	ENSSSCG00000038222
13	36876959	36876959	exonic	nonsynonymous SNV	ENSSSCG00000010681
13	59636740	59636740	exonic	nonsynonymous SNV	ENSSSCG00000010688
13	60916305	60916305	exonic	nonsynonymous SNV	ENSSSCG00000010698
13	68213577	68213577	exonic	nonsynonymous SNV	ENSSSCG00000037530
13	75704472	75704472	exonic	nonsynonymous SNV	ENSSSCG00000010717
13	90010632	90010632	exonic	nonsynonymous SNV	ENSSSCG00000032174
13	117290252	117290252	exonic	nonsynonymous SNV	ENSSSCG00000016399
13	117309598	117309598	exonic	nonsynonymous SNV	ENSSSCG00000016395
13	130501208	130501208	exonic	nonsynonymous SNV	ENSSSCG00000040172
13	132269387	132269387	exonic	nonsynonymous SNV	ENSSSCG00000015741
13	133108032	133108032	exonic	nonsynonymous SNV	ENSSSCG00000015748
13	133472575	133472575	exonic	nonsynonymous SNV	ENSSSCG00000021938
13	133868825	133868825	exonic	nonsynonymous SNV	ENSSSCG00000015898
13	136689548	136689548	exonic	nonsynonymous SNV	ENSSSCG00000025598
13	136696142	136696142	exonic	nonsynonymous SNV	ENSSSCG00000027516
13	136726657	136726657	exonic	nonsynonymous SNV	ENSSSCG00000015965
13	136726696	136726696	exonic	nonsynonymous SNV	ENSSSCG00000016017
13	136727247	136727247	exonic	nonsynonymous SNV	ENSSSCG00000060494
13	136727481	136727481	exonic	nonsynonymous SNV	ENSSSCG00000016042
13	138239941	138239941	exonic	nonsynonymous SNV	ENSSSCG00000016159
13	138544251	138544251	exonic	nonsynonymous SNV	ENSSSCG00000016177
13	140745625	140745625	exonic	nonsynonymous SNV	ENSSSCG00000016218
13	147811170	147811170	exonic	nonsynonymous SNV	ENSSSCG00000023935
13	148579051	148579051	exonic	nonsynonymous SNV	ENSSSCG00000048588
13	151484178	151484178	exonic	nonsynonymous SNV	ENSSSCG00000050847
13	158669811	158669811	exonic	nonsynonymous SNV	ENSSSCG00000009432
13	160455193	160455193	exonic	nonsynonymous SNV	ENSSSCG00000036274
13	195645147	195645147	exonic	nonsynonymous SNV	ENSSSCG00000016317
13	199940260	199940260	exonic	nonsynonymous SNV	ENSSSCG00000016335
13	200786162	200786162	exonic	nonsynonymous SNV	ENSSSCG00000016342
13	202813306	202813306	exonic	nonsynonymous SNV	ENSSSCG00000024777
13	204805453	204805453	exonic	nonsynonymous SNV	ENSSSCG00000036261
13	205250768	205250768	exonic	nonsynonymous SNV	ENSSSCG00000026842
13	207028168	207028168	exonic	nonsynonymous SNV	ENSSSCG00000016809
13	207528536	207528536	exonic	nonsynonymous SNV	ENSSSCG00000031509
14	3403522	3403522	exonic	nonsynonymous SNV	ENSSSCG00000016821

14	4354665	4354665	exonic	nonsynonymous SNV	ENSSSCG00000016843
14	7241987	7241987	exonic	nonsynonymous SNV	ENSSSCG00000016846
14	7706553	7706553	exonic	nonsynonymous SNV	ENSSSCG00000016856
14	10423719	10423719	exonic	nonsynonymous SNV	ENSSSCG00000016916
14	11169354	11169354	exonic	nonsynonymous SNV	ENSSSCG00000016956
14	12899844	12899844	exonic	nonsynonymous SNV	ENSSSCG00000037053
14	12899889	12899889	exonic	nonsynonymous SNV	ENSSSCG00000027293
14	12917822	12917822	exonic	nonsynonymous SNV	ENSSSCG00000016989
14	13728056	13728056	exonic	nonsynonymous SNV	ENSSSCG00000035845
14	13914747	13914747	exonic	nonsynonymous SNV	ENSSSCG00000017010
14	14952003	14952003	exonic	nonsynonymous SNV	ENSSSCG00000017053
14	14967285	14967285	exonic	nonsynonymous SNV	ENSSSCG00000043071
14	15305511	15305511	exonic	nonsynonymous SNV	ENSSSCG00000017076
14	15888390	15888390	exonic	nonsynonymous SNV	ENSSSCG00000017078
14	16736676	16736676	exonic	nonsynonymous SNV	ENSSSCG00000017084
14	19031314	19031314	exonic	nonsynonymous SNV	ENSSSCG00000017091
14	21108681	21108681	exonic	nonsynonymous SNV	ENSSSCG00000017109
14	21136392	21136392	exonic	nonsynonymous SNV	ENSSSCG00000003935
14	22417681	22417681	exonic	nonsynonymous SNV	ENSSSCG00000049115
14	23298701	23298701	exonic	nonsynonymous SNV	ENSSSCG00000007147
14	29871856	29871856	exonic	nonsynonymous SNV	ENSSSCG00000007146
14	29963038	29963038	exonic	nonsynonymous SNV	ENSSSCG00000007145
14	30541699	30541699	exonic	nonsynonymous SNV	ENSSSCG00000007171
14	30874440	30874440	exonic	nonsynonymous SNV	ENSSSCG00000007199
14	31289343	31289343	exonic	nonsynonymous SNV	ENSSSCG00000038183
14	38561130	38561130	exonic	nonsynonymous SNV	ENSSSCG00000007231
14	38615828	38615828	exonic	nonsynonymous SNV	ENSSSCG00000056065
14	38656446	38656446	exonic	nonsynonymous SNV	ENSSSCG00000007249
14	38828836	38828836	exonic	nonsynonymous SNV	ENSSSCG00000026137
14	38851944	38851944	exonic	nonsynonymous SNV	ENSSSCG00000024787
14	38852924	38852924	exonic	nonsynonymous SNV	ENSSSCG00000007261
14	39844713	39844713	exonic	nonsynonymous SNV	ENSSSCG00000007280
14	40098623	40098623	exonic	nonsynonymous SNV	ENSSSCG00000007288
14	40295507	40295507	exonic	nonsynonymous SNV	ENSSSCG00000023261
14	40523795	40523795	exonic	nonsynonymous SNV	ENSSSCG00000021138
14	43538998	43538998	exonic	nonsynonymous SNV	ENSSSCG00000057781
14	45924888	45924888	exonic	nonsynonymous SNV	ENSSSCG00000022714
14	49802280	49802280	exonic	nonsynonymous SNV	ENSSSCG00000021861
14	49803897	49803897	exonic	nonsynonymous SNV	ENSSSCG00000007428
14	49960140	49960140	exonic	nonsynonymous SNV	ENSSSCG00000007435
14	50256952	50256952	exonic	nonsynonymous SNV	ENSSSCG00000007458
14	50257442	50257442	exonic	nonsynonymous SNV	ENSSSCG00000058830
14	50326583	50326583	exonic	nonsynonymous SNV	ENSSSCG00000007501
14	50327021	50327021	exonic	nonsynonymous SNV	ENSSSCG00000007522

14	50327039	50327039	exonic	nonsynonymous SNV	ENSSSCG00000007526
14	50405763	50405763	exonic	nonsynonymous SNV	ENSSSCG00000007531
14	50430733	50430733	exonic	nonsynonymous SNV	ENSSSCG00000033946
14	50453733	50453733	exonic	nonsynonymous SNV	ENSSSCG00000039212
14	50586649	50586649	exonic	nonsynonymous SNV	ENSSSCG00000044842
14	51296292	51296292	exonic	nonsynonymous SNV	ENSSSCG00000055167
14	51519932	51519932	exonic	nonsynonymous SNV	ENSSSCG00000016436
14	51970632	51970632	exonic	nonsynonymous SNV	ENSSSCG00000025737
14	54862733	54862733	exonic	nonsynonymous SNV	ENSSSCG00000032249
14	54874654	54874654	exonic	nonsynonymous SNV	ENSSSCG00000016475
14	55499797	55499797	exonic	nonsynonymous SNV	ENSSSCG00000032495
14	55514997	55514997	exonic	nonsynonymous SNV	ENSSSCG00000016487
14	55807343	55807343	exonic	nonsynonymous SNV	ENSSSCG00000034639
14	55820984	55820984	exonic	nonsynonymous SNV	ENSSSCG00000028394
14	56014462	56014462	exonic	nonsynonymous SNV	ENSSSCG00000016489
14	56751401	56751401	exonic	nonsynonymous SNV	ENSSSCG00000016517
14	59204776	59204776	exonic	nonsynonymous SNV	ENSSSCG00000027118
14	61001679	61001679	exonic	nonsynonymous SNV	ENSSSCG00000016535
14	63586407	63586407	exonic	nonsynonymous SNV	ENSSSCG00000016548
14	64345024	64345024	exonic	nonsynonymous SNV	ENSSSCG00000016549
14	64727878	64727878	exonic	nonsynonymous SNV	ENSSSCG00000016579
14	65592959	65592959	exonic	nonsynonymous SNV	ENSSSCG00000016592
14	66278754	66278754	exonic	nonsynonymous SNV	ENSSSCG00000016608
14	66676106	66676106	exonic	nonsynonymous SNV	ENSSSCG00000016609
14	66678011	66678011	exonic	nonsynonymous SNV	ENSSSCG00000025602
14	66683095	66683095	exonic	nonsynonymous SNV	ENSSSCG00000016665
14	69570049	69570049	exonic	nonsynonymous SNV	ENSSSCG00000016682
14	71386895	71386895	exonic	nonsynonymous SNV	ENSSSCG00000016708
14	71512939	71512939	exonic	nonsynonymous SNV	ENSSSCG00000033313
14	71811682	71811682	exonic	nonsynonymous SNV	ENSSSCG00000016715
14	73183890	73183890	exonic	nonsynonymous SNV	ENSSSCG00000016725
14	73457091	73457091	exonic	nonsynonymous SNV	ENSSSCG00000016751
14	73691339	73691339	exonic	nonsynonymous SNV	ENSSSCG00000040257
