# Supplementary material for: Identification of Breed-Specific SNPs of Danish Large White Pig in Comparison with Four Chinese Local Pig Breed Genomes
Source: Genes (Basel). 2024 May 14;15(5):623. doi: 10.3390/genes15050623 (PMC11120843; doi:10.3390/genes15050623)
Supplement: Supplementary file 1 [file genes-15-00623-s001.zip › Supplementary Table Sl The re-sequencing information of 103 samples.pdf]

Supplementary Table Sl The re-sequencing information of 103 samples				
Sample	Clean_Reads_Num	HQ_Clean_Reads_Num(%)	Mapped_Reads	1X(%)
LW1	100145006	100012148(99.87%)	98249986-98.24%	95.96
LW2	100004854	99844212(99.84%)	93968700-94.12%	95.74
LW3	100271988	100111900(99.84%)	98883904-98.77%	96.04
LW4	100031814	99913326(99.88%)	98370766-98.46%	95.92
LW5	100223730	100069112(99.85%)	98047919-97.98%	96.05
LW6	100404970	100249566(99.85%)	99058854-98.81%	96.38
LW7	100139818	99966984(99.83%)	98844351-98.88%	96.12
LW8	100031342	99876330(99.85%)	98507766-98.63%	96.13
LW9	97829054	97671528(99.84%)	92028626-94.22%	95.6
LW10	94668472	94547416(99.87%)	93374882-98.76%	95.81
LW11	90197036	90045946(99.83%)	88420880-98.20%	95.62
LW12	100279742	100117072(99.84%)	99043417-98.93%	96.22
LW13	95032388	94863994(99.82%)	93762602-98.84%	95.86
LW14	100162980	99984746(99.82%)	98537220-98.55%	96.13
LW15	100202530	100079894(99.88%)	98777787-98.70%	96.12
LW16	100274904	100102414(99.83%)	99041479-98.94%	96.09
LW17	100295902	100143062(99.85%)	99131714-98.99%	96.19
LW18	100062858	99900212(99.84%)	98835307-98.93%	96.11
LW19	100289298	100144302(99.86%)	98869282-98.73%	96.07
LW20	100175600	100009288(99.83%)	98931110-98.92%	96.2
LW21	100263266	100084598(99.82%)	98572828-98.49%	96.03
LW22	100132912	99967470(99.83%)	95783952-95.82%	96.01
LW23	100063784	99927290(99.86%)	95001728-95.07%	95.94
LW24	100252456	100122204(99.87%)	98890364-98.77%	96.08
LW25	100085222	99925476(99.84%)	96623431-96.70%	96.16
LW26	100228870	100085938(99.86%)	98705193-98.62%	96.18
LW27	100051982	99883412(99.83%)	98623277-98.74%	96.18
LW28	100287582	100111174(99.82%)	95066708-94.96%	96.04
LW29	100301432	100163678(99.86%)	98940398-98.78%	96.05
LW30	100022644	99852342(99.83%)	98366315-98.51%	96.07
LW31	100113286	99926554(99.81%)	98777200-98.85%	96.17
LW32	98422122	98246008(99.82%)	97061748-98.79%	96.42
LW33	95998206	95868934(99.87%)	94711741-98.79%	96.09
LW34	98487222	98342860(99.85%)	97251170-98.89%	96.22
LW35	100285016	100111138(99.83%)	98354131-98.24%	96.07
LW36	100076922	99906294(99.83%)	98333502-98.43%	96.01
LW37	84600520	84478270(99.86%)	83578484-98.93%	95.27
LW38	100179980	100019420(99.84%)	98982825-98.96%	96.18
LW39	81292344	81131762(99.8%)	78319179-96.53%	94.85
LW40	95300284	95114098(99.8%)	93890283-98.71%	95.93
LW41	100022644	99851688(99.83%)	88539556-88.67%	95.49
LW42	85393862	85242978(99.82%)	79202392-92.91%	94.67

LW43	100176394	99981268(99.81%)	98961515-98.98%	96.13
AQ-10	100500000	99859148(99.36%)	98500977-98.64%	95.86
AQ-11	100500000	99862368(99.37%)	98395356-98.53%	95.65
AQ-1	100500000	99712540(99.22%)	97297406-97.58%	96.01
AQ-12	100500000	99826270(99.33%)	97292832-97.46%	95.56
AQ-13	100500000	99918804(99.42%)	86803568-86.87%	94.48
AQ-14	100500000	99833914(99.34%)	97875535-98.04%	95.86
AQ-15	100500000	99874596(99.38%)	98016190-98.14%	95.87
AQ-16	100500000	99855404(99.36%)	98422603-98.57%	95.87
AQ-17	100500000	99863802(99.37%)	98294565-98.43%	95.83
AQ-18	100500000	99852006(99.36%)	98433633-98.58%	95.79
AQ-19	100500000	99831288(99.33%)	98093599-98.26%	95.76
AQ-20	100500000	99860366(99.36%)	95896041-96.03%	95.45
AQ-21	100500000	99891772(99.39%)	98086955-98.19%	95.68
AQ-2	100500000	99746388(99.25%)	97815564-98.06%	95.43
AQ-22	100500000	99834056(99.34%)	97884927-98.05%	95.52
AQ-23	100500000	99870772(99.37%)	97894163-98.02%	95.81
AQ-24	100500000	99846464(99.35%)	98476373-98.63%	95.49
AQ-3	100500000	99818494(99.32%)	98138533-98.32%	95.69
AQ-4	100500000	99744838(99.25%)	98220416-98.47%	95.95
AQ-5	100500000	99779358(99.28%)	98341323-98.56%	95.83
AQ-6	100500000	99771034(99.27%)	96264168-96.49%	95.74
AQ-7	100500000	99783148(99.29%)	95762957-95.97%	95.99
AQ-8	100500000	99752178(99.26%)	97939052-98.18%	95.44
AQ-9	100500000	99774974(99.28%)	98073241-98.29%	96.13
DN005A	91983464	91792860(99.79%)	90887732-99.01%	95.59
DN007A	100103810	99953142(99.85%)	98949316-99.00%	95.8
DN010A	92264980	92072954(99.79%)	91227355-99.08%	95.53
DN016A	100085752	99887592(99.8%)	98968704-99.08%	95.9
DN021A	100310090	100134284(99.82%)	98905311-98.77%	95.96
DN025A	100219868	100063460(99.84%)	99178292-99.12%	96.01
DN030A	86953114	86771432(99.79%)	85850098-98.94%	95.3
DN034A	100309100	100124670(99.82%)	99243291-99.12%	95.98
DN040A	85036454	84838896(99.77%)	83997100-99.01%	95
DN052A	85204456	85045984(99.81%)	84343235-99.17%	95.32
DN054A	98018144	97810174(99.79%)	96916687-99.09%	95.87
DN056A	94424454	94255572(99.82%)	93490773-99.19%	95.93
DN061A	100055978	99854132(99.8%)	98801558-98.95%	95.98
DN087A	100251618	100029288(99.78%)	99040850-99.01%	95.89
DN093A	100133622	99941526(99.81%)	99009639-99.07%	95.89
DQZ013A	85195400	84969114(99.73%)	83668611-98.47%	93.74
DQZ018A	85050836	84888284(99.81%)	84085624-99.05%	95.19
DQZ019A	91904950	91725344(99.8%)	90811325-99.00%	95.6
DQZ020A	100295660	100086656(99.79%)	99031743-98.95%	96.02

DQZ021A	100039410	99903842(99.86%)	98898315-98.99%	95.78
DQZ023A	100200918	100038290(99.84%)	99084461-99.05%	96
DQZ024A	100313330	100117048(99.8%)	98989211-98.87%	96
DQZ028A	100011228	99830944(99.82%)	98903014-99.07%	96.03
DQZ029A	100028524	99843706(99.82%)	98895188-99.05%	96.09
DQZ030A	81479658	81307172(99.79%)	80551285-99.07%	94.87
DQZ031A	100298944	100144978(99.85%)	99190326-99.05%	95.93
DQZ032A	88733398	88589140(99.84%)	87860192-99.18%	95.35
DQZ033A	100273900	100089494(99.82%)	98726199-98.64%	96
DQZ034A	98374750	98185356(99.81%)	97308356-99.11%	95.94
DQZ052A	100006854	99858462(99.85%)	98916143-99.06%	95.98
SS-1	100500000	99656566(99.16%)	96721619-97.05%	95.75
SS-2	100500000	99525884(99.03%)	97588464-98.05%	95.67
SS-3	100500000	99671074(99.18%)	97645148-97.97%	95.91
SS-4	100500000	99518172(99.02%)	97730232-98.20%	95.66
SS-5	100500000	99611532(99.12%)	97693212-98.07%	95.78
SS-6	100500000	99550936(99.06%)	97805955-98.25%	95.91
